# Advancements in Satellite Observations of Inland and Coastal Waters: Building Towards a Global Validation Network

**DOI:** 10.3390/rs17244008

**Published:** 2025-12-12

**Authors:** Dulcinea M. Avouris, Fernanda Maciel, Samantha L. Sharp, Susanne E. Craig, Arnold G. Dekker, Courtney A. Di Vittorio, John R. Gardner, Emma Goldsmith, Juan I. Gossn, Steven R. Greb, Brice K. Grunert, Daniela Gurlin, Mahesh Jampani, Rabia Munsaf Khan, Ben Lowin, Lachlan McKinna, Colleen B. Mouw, Igor Ogashawara, Sara Rivero Calle, Wilson Salls, Joan-Albert Sánchez-Cabeza, Blake Schaeffer, Bridget N. Seegers, Jari Silander, Emily A. Smail, Menghua Wang, Jeremy Werdell

**Affiliations:** 1 U.S. Geological Survey, California Water Science Center, Sacramento, CA 95819, USA; 2 Instituto de Mecánica de los Fluidos e Ingeniería Ambiental, Facultad de Ingeniería, Universidad de la República, Montevideo 11300, Uruguay; 3 Tahoe Environmental Research Center, University of California, Davis, CA 95616, USA; 4 NASA Ames Research Center, Moffett Field, CA 94035, USA; 5 NASA Goddard Space Flight Center, Greenbelt, MD 20771, USA; 6 Goddard Earth Sciences Technology and Research II, University of Baltimore County, Baltimore, MD 21228, USA; 7 CSIRO (Commonwealth Scientific Industrial Research Organisation), Canberra, ACT 2601, Australia; 8 Engineering Department, Wake Forest University, Winston-Salem, NC 27101, USA; 9 Department of Geology and Environmental Science, University of Pittsburgh, Pittsburgh, PA 15260, USA; 10 Department of Environmental Science, Creighton University, Omaha, NE 68131, USA; 11 BAE Systems Inc. Space & Mission Systems, Boulder, CO 80301, USA; 12 European Agency for the Exploitation of Meteorological Satellites, 64295 Darmstadt, Germany; 13 Cooperative Institute for Meteorological Satellite Studies, University of Wisconsin-Madison, Madison, WI 53706, USA; 14 Department of Biological, Geological, and Environmental Sciences, Cleveland State University, Cleveland, OH 44115, USA; 15 Wisconsin Department of Natural Resources, Madison, WI 53707, USA; 16 International Water Management Institute (IWMI-CGIAR), Colombo 10120, Sri Lanka; 17 College of Environmental Science and Forestry, State University of New York, Syracuse, NY 13210, USA; 18 Skidaway Institute of Oceanography, University of Georgia, Savannah, GA 31411, USA; 19 GO2Q PTY LTD, North Rocks, NSW 2151, Australia; 20 Graduate School of Oceanography, University of Rhode Island, Narragansett, RI 02882, USA; 21 Department of Plankton and Microbial Ecology, Leibniz Institute of Freshwater Ecology and Inland Fisheries, 16775 Stechlin, Germany; 22 U.S. Geological Survey, New York Water Science Center, Troy, NY 12180, USA; 23 Unidad Académica Mazatlán, Instituto de Ciencias del Mar y Limnología, Universidad Nacional Autónoma de México, Mazatlán 82040, Mexico; 24 Office of Research and Development, U.S. Environmental Protection Agency, Durham, NC 27709, USA; 25 Finnish Environment Institute, 00790 Helsinki, Finland; 26 NOAA/NESDIS/Office of Satellite and Product Operations, College Park, MD 20740, USA; 27 NOAA Center for Satellite Applications and Research, College Park, MD 20740, USA

**Keywords:** water quality, remote sensing, validation, monitoring, field measurements

## Abstract

The use of satellite-based remote sensing imagery for water quality monitoring of inland and coastal waters has become widespread over the last few decades, with the expansion of, and investment in, operational Earth-observing missions. Satellite-based sensors are uniquely suited to provide synoptic, system-wide water quality parameter estimates that supplement traditional field-based sampling methods. The remote sensing of water quality parameter estimates is particularly valuable in systems with high temporal and spatial variability, as well as in areas that are difficult to access, or where agencies lack funding for routine monitoring. However, optically complex inland and coastal waters pose additional challenges for developing robust remote sensing retrieval models for optical properties and water quality parameters. One of the biggest challenges is collecting high quality field measurements that are used to calibrate and validate the retrieval algorithms. Here, we present the current status of satellite missions, field methods that include instruments used and commonly measured parameters, and repositories of historical field data that are relevant to inland and coastal water studies. We then present data requirements for model validation and highlight gaps in validation coverage. Finally, we provide considerations for future field campaigns to improve coordination with remote sensing data collection and ensure that field data is well suited for use in model or algorithm development.

## 1. Introduction

### 1.1. Why Are Inland and Coastal Waters Important?

Inland and coastal waters account for 0.0065% and 8–10% of all surface water on Earth, respectively [1,2], but they provide invaluable environmental services and socioeconomic benefits that support our modern society and day-to-day life. Inland water bodies such as rivers, canals, lakes, and reservoirs often serve as drinking water supplies, enable navigation, provide recreational opportunities, support fisheries and irrigated agriculture, and are of cultural significance. Coastal water bodies are at the interface between the land and sea in bays, inlets, coves, estuaries, and harbors; they often support fisheries, serve as storm barriers, and host recreational and cultural activities. Inland and coastal water ecosystems provide many environmental benefits; they naturally filter pollutants and nutrients, regulate climate, and serve as biodiversity hotspots [3]. Societally, they influence cultures and human health and well-being [4].

As inland and coastal waters have been leveraged for economic development, their spatial extent [5,6], biogeochemistry [7], and ecosystem functions [3,8] have been altered to varying degrees. These water bodies are now arguably the most imperiled ecosystems on Earth [9]. Rapid changes are often reflected in global water quality assessments that show increased suspended particulate matter (SPM) loads in rivers [10], higher chlorophyll-a pigment concentration (Chl *a*) in coastal waters [11], more frequent observations of harmful algal blooms (HABs) [12] and invasive species [13], and decline in coverage of submerged aquatic vegetation in lakes [14,15] and seagrass in coastal waters [16]. Further, water quality degradation is impacting freshwater species, including mammals, amphibians, birds, reptiles, and fish, which have declined in population by an average of 83% from 1970 to 2018 according to the World Wildlife Fund (WWF) Living Planet Index [17]. Inland and coastal water systems are generally warming and becoming more eutrophic due to climate change and increased nutrient loads, both of which can further increase the occurrence of HABs in some locations [18], devastating fisheries and forcing managers to close recreational areas. A global estimate of the annual economic losses related to HAB events was around $10 billion U.S. dollars in 2014 [19].

### 1.2. Advantages and Disadvantages of Remote Sensing as a Tool

Optical satellite-based remote sensing of aquatic targets (herein referred to as remote sensing) has emerged as a powerful tool for environmental studies because it provides unique advantages over in situ sampling techniques. By using satellite-based sensors, remote sensing allows for consistent repeat observations through space and time on scales unattainable by conventional ground- or aircraft-based sampling, enabling comprehensive global-scale assessments [20]. Specifically, remote sensing facilitates landscape-scale imagery of inland and coastal areas, potentially overcoming spatial and temporal biases that remain unavoidable with traditional in situ sampling methods [21]. Furthermore, historic satellite data records can be periodically reprocessed as remote sensing methods emerge or improve, offering a “time machine” for environmental monitoring. This level of data collection is valuable to extract whole-system processes from observed patterns, pinpoint hotspots of change, and reduce bias in sampling programs.

One advantage of remote sensing is its accessibility for hard-to-reach and potentially hazardous locations. This accessibility is enhanced by the temporal repeat cycle, providing valuable data that would otherwise be missed [22]. Remote sensing is cost-effective to the user, offering a complement to in situ sampling techniques, which are time-consuming, labor-intensive, and limited in spatial coverage [21–23]. Furthermore, remote sensing has the potential to produce datasets where no in situ data existed previously, such as regions that may not have resources available for sampling and analysis [20].

While remote sensing offers significant advantages, there are limitations, such as: restrictions on what aquatic variables can be measured [21,24]; impacts from clouds, sun glint, bottom reflectance, and adjacency effects from surrounding terrestrial areas [21,22,24,25]; the need for rigorous processing, including atmospheric correction, to generate good quality aquatic data products [22,25]; and being limited to the first optical depth. Additionally, for remote sensing data to provide meaningful measurements, calibration of the sensors and performance assessments of the derived data products are essential [25]. Finally, spatial resolution (pixel size), spectral resolution (channel wavelength centers and widths), and temporal resolution (data collection frequency) may be limitations for remote sensing usage. Despite these limitations, remote sensing provides a valuable complement to in situ measurement programs given its frequent, repeat, large-scale global sampling.

### 1.3. Gaining User Trust and the Role of Validation

The availability and utility of remote sensing data products are continuing to expand. However, the adoption of these data products requires trust on the part of end users, especially for water quality parameters derived from remote sensing. Often, users expect data and products delivered by government agencies and other trusted organizations to be accurate and precise. When users compare values reported in a remote sensing product with their own in situ data and see a mismatch, they can lose confidence in remote sensing products. A data product may be precise in a certain geographic region or water type but be identified as having unknown or limited performance elsewhere due to a lack of validation data and/or other processing challenges. Developing and maintaining trust for water quality products derived from remote sensing requires a transdisciplinary approach, time, continued engagement, and communication between agencies and other trusted organizations with end-users [26–28]. As a result, end-user requirements should drive required product performance and acceptable levels of uncertainty for remote sensing products. This challenges the assignment of a unified and ubiquitous framework for defining what is an “acceptable” remote sensing retrieval. For example, a categorical data product that indicates the presence or absence of a water quality issue can be meaningful for some users and requires a different approach for validation than a precise quantitative estimate of a water quality parameter, measured as a continuous variable, that another user might require.

Overall, there is increasing interest in using satellite remote sensing to help manage and monitor inland and coastal waters, and for scientific research in spatiotemporal changes in water bodies. However, established inland and coastal water validation sites are often limited and do not encompass global variability, limiting the number of well-characterized products across a few selected dynamic ranges and retrieval conditions. The global network discussed here does not represent a tangible network, managed by a single entity, but rather an advance towards compatible in situ measurements, that together constitute global coverage of data available for remote sensing model validation. Increased validation efforts for inland and coastal waters are thus essential to providing acceptable products that meet the needs of end users.

### 1.4. Publication Aims and Objectives

The idea for this paper was conceived at the National Aeronautics and Space Administration (NASA) Workshop on the Validation of Satellite-derived Optical and Water Quality Parameters for Coastal and Inland Waters, held at the University of Wisconsin-Madison, 7–9 June 2022; a decade after a similar workshop on Coastal and Inland Remote Sensing [29]. The workshop convened with the purpose of sharing knowledge about the current state of the science, particularly the validation of satellite-derived optical and water quality parameters for inland and coastal waters. This paper focuses on what is needed from existing in situ measurements and protocols, identifies current in situ measurement databases and gaps for future improvements, and the effort required for validating coastal and inland water satellite imagery. The goal is to better utilize data products from current and upcoming satellite missions for optically complex water bodies. As such, the specific goals of this paper are to: (1) summarize the state of the science of satellite remote sensing products for coastal and inland waters; (2) identify validation needs for remote sensing reflectance products and derived water quality parameters; and (3) identify improvements in validation protocols, optimized for inland and coastal water conditions.

Our primarily intended audience is the scientific community and stakeholders involved in water quality monitoring and management. As such, the technical terminology that refers to remote sensing was carefully defined, and several sections may have information that is well-known by remote sensing experts and experienced users. Nevertheless, we aimed to cover all aspects relevant to validating satellite products, including less targeted parameters and aspects related to sensor technologies. We believe that the involvement of a broad audience is necessary to successfully advance the development of robust remote sensing models for quality by presenting the current state of the science and gaps and to ultimately improve the usefulness of satellite-derived products.

## 2. State of the Science

### 2.1. Advancements Since 2015

Community workshops held in 2012 and 2022 brought together a diverse community of science developers, users, and managers to identify the need to advance remote sensing in coastal and inland water bodies. The 2012 workshop [29] focused on the four fundamental elements of aquatic satellite remote sensing: mission capability, algorithm development, in situ observations, and operational capacity [25]. Primary challenges and recommendations for each of the four areas were identified at that time and are briefly summarized here.

Mission capability—the continued demand for geostationary and polar orbiting missions with appropriate sensitivity, spectral, spatial, and temporal scales, with expanded spectral resolution, was identified due to the lack of on-orbit missions focused on the scales of variability of coastal and inland water bodies at the time.

Algorithm development—the desire to focus on product continuity rather than algorithm continuity and the requirement of intercomparison exercises to identify algorithm strengths and limitations, resulting in consolidation of algorithm frameworks in optically diverse regions, were identified.

In situ observations—the opportunity to support hyperspectral mission instrumentation gaps, along with the demand to update protocols to account for a broad dynamic range of optical and biogeochemical variability, and minimum observations were recommended for field observations to have the most significant impact in validation efforts. Clear, consistent, and coordinated data sharing policies between agencies with centralized publicly available data repositories to ensure access to consistent, high-quality data, regardless of funding source, was recommended to enable meeting broad user group requirements.

Operational capacity—the most significant challenges were identified as the mismatch between the information supplied by remote sensing data and the information desired by end users; the user’s ability to work with software, data volumes, and file types; and the user’s ability to identify products with the least uncertainty for given applications. The following suggestions were identified to reduce this mismatch: expand training opportunities; establish a panel of rotating experts that could provide advice on fit-for-purpose solutions; expand accompanying decision support tools and infrastructure developed for and in consultation with the user community; ensure free, publicly available, and timely access to all data streams; and develop a user-driven community of practice.

A decade later, significant strides in mission capability have been accomplished or are planned for the near future (see Section 2.2). Many instrumentation advancements have been accomplished (e.g., the availability of hyperspectral backscattering instruments; see Section 2.3). Many algorithm advancements have emerged, including frameworks focusing on optical water types as the basis for deciding the most appropriate algorithm to apply (e.g., [30]). In addition, technological advances in computing power and methods have allowed machine learning and data driven models to be applied to remote sensing data, improving model performance for optical water quality parameters [31–36]. The emergence of these powerful modeling tools also opens the door for development of optical proxy models for non-optical parameters such as nutrients and contaminants (PFAS, mercury, etc.). Machine learning frameworks still require in situ validation datasets, however, and coastal and inland in situ observations from around the globe are lacking standardization and remain in disparate databases with a broad range of observations based on the observer’s goals, skills, and accessibility to various instrumentations. In this way, elements from the in situ observation and operational capacity recommendations from [25] have not yet been fully realized. Thus, validation of satellite-derived and optical water quality parameters for coastal and inland waters remains a data gap and area of interest for continued community investment, which was the focus of the 2022 workshop.

### 2.2. Current & Upcoming Missions

Satellite-based remote sensing missions have been developed and launched by multiple countries. The NASA, National Oceanic and Atmospheric Administration (NOAA), and United States Geological Survey (USGS) in the United States of America, and the European Space Agency (ESA) and European Organization for the Exploitation of Meteorological Satellites (EUMETSAT) in the European Union (EU) have led the most missions, but China, India, Japan, and South Korea have also launched operational Earth-observing sensors. Currently, operational sensors span a range of spatial, temporal, and spectral resolutions. These missions can be generally separated into high (<100 m) and moderate (>100 m) spatial resolution subsets. Missions can also be separated by temporal resolution, with repeat visitation ranges of 1–16 days, with some geostationary satellites offering multiple retrievals per day for specific areas of the Earth. All but two of the current missions are multispectral sensors, with some having spectral bands in regions optimized for aquatic retrievals. Details of operational (as of 21 May 2025) missions can be found in Table 1.

These missions (Table 1) demonstrate advancements that include hyperspectral resolution sensors, increased spatial resolutions, and missions dedicated to coastal and inland waters. Mouw et al. [25] called for sensor missions with expanded spectral resolution, increased sensitivity, and increased spatial resolution while maintaining a short revisit period (temporal resolution). These requirements are important for monitoring essential biodiversity variables [37]. Some of these recommendations have been realized in the Sentinel 2 and Sentinel 3 missions. Both the NASA Phytoplankton, Aerosol, Cloud, ocean Ecosystem (PACE) and the Italian Space Agency (ASI) Hyperspectral Precursor of the Application Mission (PRISMA) missions carry hyperspectral sensors. While PACE (launched on 8 February 2024) was designed for ocean color applications with a 1 km pixel size and a 1–2 day revisit time, PRISMA’s sensor (launched on 22 March 2019) was optimized for coastal and inland waters (as well as vegetation) with 30 m spatial resolution, but it operates in a “user driven” targeting mode with a re-targeting period of up to 7 days and a response time of up to 14 days. The Environmental Mapping and Analysis Program (EnMAP) mission provides hyperspectral data at a 30 m resolution every 27 days.

Other novel color satellite missions to be mentioned are the SeaHawk-1 (multispectral, 120 m resolution, launched in 2018 and operational until November 2023) and the Hyper-Spectral Small Satellite for Ocean Observation 1 (HYPSO-1; hyperspectral, 140 m resolution, launched in 2022) CubeSat missions, which have been led by academic institutions mostly as proof-of-concept missions. SeaHawk-1 was funded by the Gordon and Betty Moore Foundation and supported by a Space Act Agreement with NASA; it produced a global coverage of inland and coastal waters, and its data is freely available through NASA’s ocean color website. On the high end of spatial resolution, it is relevant to mention the PlanetScope satellite constellation, which consists of multiple launches of individual CubeSats (Doves and SuperDoves, multispectral, 3–4 m resolution, daily revisit time). PlanetScope mission’s data is freely available for scientific research and application development through ESA’s website upon submission and acceptance of a project proposal.

Upcoming missions that will likely be important for inland and coastal water applications include the NASA Surface Biology and Geology (SBG) mission (in planning stages, possible launch in 2028) that will likely carry a hyperspectral sensor with a 30 m pixel size and a 1–2 week revisit time, the expansion of the Landsat suite of satellites with Landsat Next (expected launch 2030), the Geostationary Littoral Imaging and Monitoring Radiometer (GLIMR, expected launch 2026–2027), and the NOAA satellite mission Geostationary Extended Observations (GeoXO, expected launch 2032). These upcoming missions could greatly expand the scientific possibilities for remote sensing of inland and coastal waters. They also represent the increased focus on Earth observation data that is optimized for water targets. However, the demand remains for additional missions with spectral resolution and sensitivity to resolve optically complex water bodies with small enough spatial resolution for inland water bodies.

### 2.3. Existing In Situ Sensor Technology

In situ sensor technology has advanced considerably over the last two decades. Sensors are becoming smaller, modular, more user-friendly, and more accurate. In situ sensors can now measure more parameters including inherent and apparent optical properties (defined below) and a wide range of water quality parameters. Traditionally, water samples are manually collected in the field, and the water properties are measured using laboratory analysis techniques. This approach can be costly and time-consuming. In situ sensors also have costs including regular maintenance, and a learning curve associated with their use; however, these are generally lower than traditional sampling techniques. Field sensors increasingly complement these traditional sampling and laboratory analysis approaches. In addition to their use in specific sampling campaigns, field sensors can be deployed to measure water properties continuously, providing data in real time in some cases. Autonomously deployed sensors can increase the quantity of data for satellite data product validation, reduce data collection efforts, speed up data delivery, and provide high-frequency measurements. Continuous sampling with these instruments can resolve temporal variability in water conditions that satellite sensors cannot capture.

#### 2.3.1. IOPs, AOPs, and Water Quality Attributes

Inherent optical properties (IOPs) of water describe how light interacts with an aquatic medium and are wavelength dependent. IOPs are independent of incident illumination conditions (e.g., clouds or time of day). They fall into two main categories: absorption and scattering [38,39]. IOPs can be used to describe interactions of sunlight with dissolved and particulate matter via absorption and scattering effects. Sunlight can be absorbed by constituents in the water column or scattered in different directions. Bulk IOPs, the total absorption coefficient (*a*) and the total scattering coefficient (*b*), describe the absorption and scattering effects of all the constituents in the water column, including water itself. These bulk IOPs can be broken down into sub-components, such as chromophoric dissolved organic matter (CDOM), pigmented algal particles, and non-algal particles (NAPs, e.g., sediment & detritus), which can be operationally relevant. The beam attenuation coefficient (*c*) is also an IOP that measures how sunlight dissipates in the water column and is the sum of *a* and *b*. Another commonly used IOP is the backscattering coefficient (*b_b_*) that describes the portion of light scattered into a backwards-facing hemisphere relative to the path of an incoming beam of light.

The apparent optical properties (AOPs) of water depend upon the IOPs and the illumination conditions and vary by wavelength. These properties are used to gain insights into the optical constituents of a waterbody [38]. Commonly measured AOPs include reflectance and diffuse attenuation coefficients (*K*-functions), which describe the rate at which light intensity decreases with depth. Reflectance is a measure of the fraction of the incoming sunlight (downwelling irradiance, *E*_d_) relative to the amount that is emerging from within the water column and can be based on upwelling irradiances or radiances. Remote sensing reflectance (*R*_rs_) is often used in aquatic remote sensing. It is derived through normalizing water-leaving radiance (*L*_w_) to the incoming sunlight reaching the water surface (*E*_d_). It can be measured in the field using radiometric sensors that measure upwelling radiance and downwelling irradiance and is often used for comparisons with equivalent satellite data products. Diffuse attenuation coefficients (upwelling and downwelling) describe the amount of light dissipated along a vertical pathlength in the water column and can be measured directly using profiling radiometric sensors (see Section 2.3.3). The diffuse attenuation coefficient of downwelling irradiance (*K*_d_), and more specifically the diffuse attenuation coefficient of photosynthetically active radiation (*K*_PAR_), is a standard measure in environmental assessments, such as in the United States Environmental Protection Agency (EPA) National Coastal Condition Assessment (NCCA).

Additionally, water quality attributes are commonly derived from satellite *R*_rs_ using retrieval algorithms or models [21,22,24,25]. These include Secchi disk depth, turbidity (including related attributes of total suspended solids (TSS) and SPM), Chl *a*, accessory algal pigments concentrations such as phycocyanin (PC) and phycoerythrin (PE), and CDOM, among others. The acronyms and symbols used in this review are summarized in Table 2.

#### 2.3.2. IOP Measurements

IOPs, such as absorption, scattering, and beam attenuation coefficients can be measured using a variety of sensors and methods (Supplementary Table S1). Measurement units can vary depending on the property, but typically absorption and scattering coefficients are expressed in units of per meter (m^−1^), or in units of per meter per steradian (m^−1^ sr^−1^). Some sensors can perform hyperspectral measurements, while others measure at single or multiple wavelengths.

One of the most commonly used instruments to measure absorption and beam attenuation in the field is an absorption and attenuation meter (ac-meter) (Table S1). This type of instrument has two flow tubes, one with a matte black internal surface for measuring *c*, and the other fitted with a reflective quartz sleeve for measuring *a*. An ac-meter measures the absorption coefficient of suspended particles plus CDOM (*a*_p_ + *a*_CDOM_), or CDOM (*a*_CDOM_) alone when a 0.2 micrometer (μm) filter is attached. Another type of absorption sensor is known as integrating cavity absorption meters (ICAMs). In ICAMs, water is pumped through a flow-through point source-integrating cavity (Table S1). The ac-meters can also measure *b* by computing the difference between the measurements of *c* and *a*. Another option to measure *b* (and *b*_b_) is to use multi-angle volume-scattering function (VSF) meters (Table S1), while fixed-angle VSF-meters, also known as backscattering sensors (Table S1), are more routinely used to determine *b*_b_ [40].

IOPs sensors require careful calibration, correction, and field deployment procedures. Absolute calibration standard reference materials are needed, such as pure water, certified absorption standards (e.g., nigrosin), calibrated scattering standards, or non-absorbing scattering materials (e.g., polystyrene microbeads). Absorption and scattering are temperature and salinity dependent, and ideally corrected using simultaneous temperature and salinity measurements and well documented correction equations [41–43]. Furthermore, many IOPs in situ sensors have been developed for oceanic waters, posing limitations in optically complex waters, and requiring corrections or adaptation of sensor technologies, as detailed in Section 3.3. Alternatively, particulate matter absorption can be measured spectrophotometrically in the laboratory, using filter pads [44].

#### 2.3.3. AOP Measurements

*R*_rs_ can be estimated from field measurements of upwelling radiance and downwelling irradiance made at a sampling location using field radiometric instruments (Supplementary Table S2). Radiometric measurements are collected through manually or automatically operated sensors. Measurement approaches can be broadly divided into two categories: (1) above-water and (2) in-water radiometry, where upwelling radiance and downwelling irradiance are measured above or in the water [45,46]. A special case of above-water radiometry is adding skylight-blocking techniques for shielding the sensor from reflected sky radiance to measure *L*_w_ near the water surface [47]. Sensors can be hand-held and pointed in the appropriate directions to measure the radiometric variables of interest or installed on a floating frame, boat, fixed platform, or profiler. Radiances are expressed in watts per square meter per steradian (W m^−2^ sr^−1^), whereas irradiances are expressed in watts per square meter (W m^−2^). *R*_rs_ is, therefore, reported in units of per steradian (sr^−1^).

*K*_d_ can be estimated from field measurements of downwelling irradiance made at different depths by profiling field spectroradiometers; whereas *K*_PAR_ is more commonly measured in the field using PAR sensors that quantify the spectrally integrated photosynthetic flux density of photons (in the range 400–700 nm at different depths) (Table S2). Log-transformed irradiance (or PAR) typically decreases linearly as a function of depth, and the slope of this linear relationship is *K*_d_ (or *K*_PAR_). We note, however, that *K_PAR_* is not a depth independent parameter and can lead to erroneous estimates of PAR propagated to depth [48]. Measurement approaches are limited to in-water radiometry, and irradiance measurements are often taken at the same depths as other water column indicators. Sensors can be added to any profiler to accommodate their size and type. Diffuse attenuation coefficients are reported per meter (m^−1^).

#### 2.3.4. Water Quality Measurements

In situ water quality sensors can be used to directly relate water quality attributes to satellite remote sensing reflectance and/or be used in combination with the aforementioned IOPs and AOPs to validate bio-optical models (see Section 2.5 for more details on the validation process). Near real-time and real-time water quality in situ measurements can complement discrete sampling for validating satellite data products, such as when developing real-time water quality monitoring and forecasting systems (e.g., [49]). In situ sensors (Supplementary Table S3) can measure optical water quality attributes such as turbidity, Chl *a*, accessory algal pigments, and CDOM, based on the absorbance, scattering, or fluorescence principles associated with a given water quality parameter.

Turbidity can be measured using scattering-based in situ sensors (Table S3). Common units include nephelometric turbidity ratio units (NTU) and formazin nephelometric units (FNU). Scattering-based turbidity sensors are now so robust and accurate that low-cost, do-it-yourself turbidity sensors rival commercial sensors in accuracy and usability [50,51]. To obtain physical units of mass/volume concentration (e.g., milligrams per liter (mg/L)), field samples can be analyzed in a laboratory for SPM concentration, typically by filtering the water and weighing the dried contents on the filter. Turbidity sensors can be related to laboratory measurements of SPM due to the strong correlation between SPM and turbidity.

Chl *a* and accessory algal pigments, such as PC and PE, are typically measured using fluorescence-based in situ sensors (Table S3). Fluorescence is an inelastic scattering process that occurs when a material is irradiated with light of a certain wavelength, which is absorbed and re-emitted at a longer wavelength. Most commercial Chl *a* fluorescence sensors output concentrations in units of micrograms per liter (μg/L) using laboratory-based calibrations with Chl *a* standards, but they may require site-specific calibration using field samples and ambient temperature corrections [52].

CDOM is a complex mixture of organic molecules and is operationally defined as the optically active components dissolved in water that pass through a 0.2 μm pore-size filter [53]. CDOM can be characterized as a water quality attribute using a range of measurement methods (Table S3). The absorption coefficient of CDOM (^*a*^_CDOM_) at a specific wavelength (e.g., 440 nm) is an IOP. In situ CDOM sensors typically use fluorescence with units of relative fluorescence units (RFU) or quinine sulfate dihydrate units (QSU) and require site-specific calibration using field samples and ambient temperature corrections [54]. Absorbance-based measurements using an in situ or laboratory spectrophotometer are also common, traditionally measuring absorbance at one or more wavelength bands and reporting the derived absorption coefficient in units of m^−1^ together with its wavelength (such as ^*a*^_CDOM_(440)). For CDOM absorption measurements, water samples must be passed through a 0.2 μm pore-size filter. This can be performed for both laboratory and in situ sensors.

### 2.4. Existing Databases

In anticipation of the continued growth of inland and coastal water quality monitoring approaches, many databases may be considered for validating satellite-derived water quality attributes. In situ data are essential for advancing bio-optical models, including their development and validation, and building future capabilities. Ideally, databases would consist of well-documented and quality-controlled IOPs and AOPs across wide geographic areas and over large dynamic ranges. However, very few datasets are compiled with the purpose of validating bio-optical algorithms and the resulting satellite-derived water quality attributes. While well-established methods for validation exist in ocean settings [55], new methods and alternative options may need to be established in inland and coastal waters [56]. This section briefly describes several known databases that are likely to advance such algorithms for inland and coastal waters. These databases are publicly aggregated and linked through the Group on Earth Observations (GEO) AquaWatch website (Water Quality Database Inventory–AquaWatch (https://www.geoaquawatch.org/water-quality-database-inventory/ accessed on 1 July 2024)) [57]. These databases have varying levels of quality control parameters that are applied to the datasets that are included. This information is typically available on the database website, and should be taken into consideration when leveraging these resources.

Data access policies range from restricted access in the University of Stirling’s Lake Bio-optical Measurements and Matchup Data for Remote Sensing (LIMNADES) database to mixed access at the Commonwealth Scientific and Industrial Research Organisation’s (CSIRO) Data Access Portal (DAP), to a majority of publicly accessible databases such as the Water Quality Portal’s (WQP) underlying databases, Global Lake Ecological Observatory Network’s (GLEON) LakeBase, and Global Water Quality Database (GEMStat). Temporal coverage within databases is broad, from 1844 through the present day, with temporal intervals and date ranges varying by location. Spatial coverage of these databases is typically more aligned with database funding efforts, and ranges in scale from global, such as in NASA’s SeaWiFS Bio-optical Archive and Storage System (SeaBASS), to regional, with the European Environment Agency’s (EEA) Waterbase and the Latin American Research Network of Marine-Coastal Stressors in Latin America and the Caribbean’s (REMARCO) similarly named platform, to national, such as in the Australian National Collaborative Research Infrastructure Strategy (NCRIS) enabled Integrated Marine Observing System (IMOS) and the Gordon Foundation initiated DataStream. Data access is handled universally through URL electronic records access, and the ability to contribute to these databases varies.

### 2.5. The Validation Process

**Figure 1.**
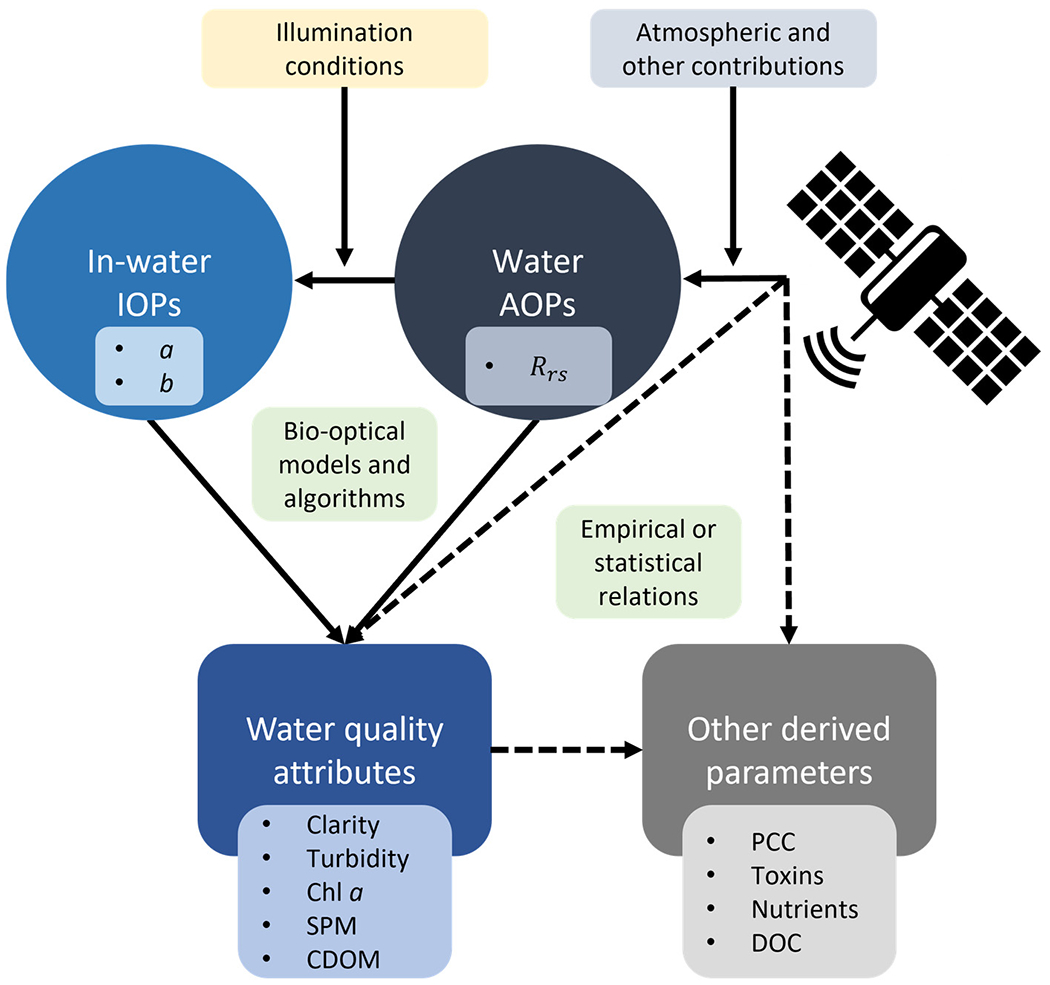
Schematic diagram of inputs for bio-optical models and algorithms to obtain water quality parameters from satellite remote sensing: optical satellite sensor measurements are dependent on in-water inherent optical properties (IOPs), illumination conditions, and atmospheric and other contributions (e.g., adjacency effects, sun glint, and reflected sky radiance). Water apparent optical properties (AOPs), and particularly remote sensing reflectance (*R*_rs_), depend on in-water IOPs and the illumination conditions. Bio-optical models and algorithms can relate water quality attributes to IOPs or AOPs. More indirect derivations, which often involve statistical relationships, are indicated with dashed arrows. *a*, absorption coefficient; *b*, scattering coefficient; Chl *a*, chlorophyll *a*; SPM, suspended particulate matter; CDOM, chromophoric dissolved organic matter; PCC, phytoplankton community composition; DOC, dissolved organic carbon.

Water quality attributes can be analytically related to satellite measurements through AOPs, which in turn depend on in-water IOPs, through different types of algorithms and bio-optical models (Figure 1). Other satellite retrievals of interest can include, but are not limited to, phytoplankton community composition (PCC), nutrients, toxins (e.g., produced by cyanobacteria), and dissolved organic carbon (DOC), which are often indirect derivations that involve statistical relationships. The validation process of these products can focus on different relations depicted in Figure 1. Frequently used approaches are: (1) validating field water quality parameters directly against their satellite-retrieved matchups; and (2a) validating in situ radiometric measurements, usually *R_rs_*, against their satellite-retrieved matchups, followed by (2b) validating field water quality parameters against their estimations derived from simultaneous in situ radiometric measurements.

Approach (1) is desirable for end-user applications, as it provides a bulk estimation of the related errors and uncertainties. However, it does not provide information regarding the source of these uncertainties, while approach (2) provides further insight into this matter. For example, approach (2a) allows errors or uncertainties to be distinguished that might be generated by the atmospheric correction process of satellite data (Figure 1), while (2b) can be useful for refining algorithms or models without the uncertainties related to the atmosphere, adjacency effects, and other contributions. Each approach has different needs regarding in situ measurements (Section 3.1), and approach (2) may also require reconciling hyperspectral field radiometric measurements with multispectral satellite bands. The mathematically correct approach involves the convolution of radiance and irradiance measurements with satellite spectral response functions before computing *R*_rs_ [58].

Previous studies have used both linear and log-transformed metrics to compare estimates (*E*) against in situ measurements (*M*) to evaluate the quality of remote sensing retrievals. Among typically used linear metrics are the mean absolute error (MAE), the mean error or bias (ME), the root mean square error (RMSE), and the slope of the linear regression (S). Performance metrics are often referred to as “differences” instead of “errors” (e.g., RMSD instead of RMSE), acknowledging that in situ measurements are not perfect ground truths. The ME and MAE can also be computed as relative errors (in percentages), named the mean percentage error (MPE) and the mean absolute percentage error (MAPE). ME, MPE, MAE, MAPE, and RMSE can be computed as follows:

(1) $$\text{ME}=\frac{1}{N}\sum_{i=1}^{N}\left(E_{i}-M_{i}\right),\text{and MPE}=\frac{1}{N}\sum_{i=1}^{N}\left(\frac{E_{i}-M_{i}}{M_{i}}\right)\times 100$$

(2) $$\text{MAE}=\frac{1}{N}\sum_{i=1}^{N}\left|E_{i}-M_{i}\right|,\text{and MAPE}=\frac{1}{N}\sum_{i=1}^{N}\left(\frac{\left|E_{i}-M_{i}\right|}{M_{i}}\right)\times 100$$

(3) $$\text{RMSE}=\sqrt{\frac{1}{N}\sum_{i=1}^{N}{\left(E_{i}-M_{i}\right)}^{2}}$$

Values closer to 0 of metrics in Equations (1)–(3) indicate better performance. To compute S, the RMSE is generally minimized, and values closer to 1 indicate better performance. MAE, MAPE, and RMSE give an idea of the accuracy of the estimations, while ME and MPE reveal a general bias, with positive values indicating overestimation and negative values underestimation. In some studies, the median (Md) instead of the mean is used in Equation (2) to compute the median absolute percentage error (MdAPE; [59]), to reduce the impact of outliers in the metric result. When the evaluated parameters expand within several orders of magnitude (e.g., Chl *a*), log-transformed metrics might be more appropriate. Among these types of metrics are the root mean squared log-difference (RMSLE; Equation (4)), and the versions of ME, MPE, MAE, and MA PE computed in log-space, as shown in the example of Equation (5) for MAE.


(4) $$\text{RMSLE}=\sqrt{\frac{1}{N}\sum_{i=1}^{N}{\left(log_{10}\left(E_{i}\right)-log_{10}\left(M_{i}\right)\right)}^{2}}$$

(5) $${\text{MAE}}_{\text{log–space}}=10^{Y},\;\text{where}\;Y=\frac{1}{N}\sum_{i=1}^{N}\left|log_{10}\left(E_{i}\right)-log_{10}\left(M_{i}\right)\right|$$

In addition, a recent study [60] recommends the median symmetric accuracy (*ζ*) and the signed systematic percentage bias (SSPB) as robust and resistant metrics to evaluate performance; they can be computed as:

(6) $$\zeta =\left[e^{X}-1\right]\times 100,\;\text{where}\;X=median\left(\left|log_{e}\left(\frac{E_{i}}{M_{i}}\right)\right|\right)$$

(7) $$\text{SSPB}=sign(X)\times \left[e^{\left|Z\right|}-1\right]\times 100,\;\text{where}\;Z=median\left(log_{e}\left(\frac{E_{i}}{M_{i}}\right)\right)$$

Metrics ζ and SSPB equally penalize over- and under-estimations, and they mitigate the effects of outliers while still maintaining interpretability, as they are both reported as a percentage [60]. Nevertheless, metrics in Equations (1) through (5) have been widely used in previous works (e.g., [61,62]) and might be necessary to compute if comparability is desired.

## 3. Outstanding Validation Gaps

Much work has been performed to develop remote sensing models for water quality parameters in inland and coastal waters, including Chl *a*, turbidity, CDOM, and *K*_d_ (e.g., [61–72]); however, this work is still on-going, as many of the current models are developed for limited geographical areas or water types. Therefore, it is important to expand the capabilities to characterize all optically active water constituents to fully understand the dynamics of aquatic systems and their associated reflectance spectra. Ideally, in situ measurements for the characterization of an aquatic system will include quantifying IOPs, direct measurements of water quality parameters, including measurements of phytoplankton pigments, nutrients, contaminants, and analyzing PCC.

The ongoing challenge for bio-optical model development in coastal and inland water bodies is the limited number of matched data pairs of water reflectance and other field data. These measurements are necessary to understand the optical complexity of the freshwater and coastal systems, and to develop improved bio-optical models. The following sections detail the outstanding validation gaps of different parameters (Section 3.1), the remaining challenges to transition from open ocean to inland and coastal water validation protocols (Section 3.2), the limitations of field sensor technologies to measure less targeted products—particularly IOPs—in optically complex waters (Section 3.3), and the current validation gaps in terms of parameters, geography, and user engagement (Section 3.4).

### 3.1. Validation Gaps for R_rs_ and Water Quality Parameters

Validation of satellite-derived products (e.g., water quality parameters) with field data is critical to support the use of current and upcoming satellite missions for scientific studies and water quality management. Because many bio-optical models and algorithms are developed using the relationship between satellite-based measurements of *R*_rs_ and field measurements, their calibration and validation depend on both high-quality measurements of *R*_rs_ from satellites and high-quality field measurements. This section describes current validation needs, specifically the requirements for reliable field and laboratory measurements of AOPs, IOPs, and associated parameters within the water column (Figure 1). We focus on *R*_rs_, water clarity (Secchi depth and *K*_d_ at 490 nm (*K*_d_(490)), turbidity, SPM, Chl *a*, and ^*a*^_CDOM_. Field measurements needs of *R*_rs_ and these parameters for validation can differ, as detailed below in Sections 3.1.1 and 3.1.2.

#### 3.1.1. Remote Sensing Reflectance (R_rs_)

The primary field measurement method for calibration and validation of *R*_rs_ is above- or in-water radiometry (henceforth radiometry). Efforts have been made to develop radiometric networks (e.g., Aerosol Robotic Network—Ocean Color (AERONET-OC), HYPERNETS) that support calibration and validation of satellite-based *R*_rs_ measurements (e.g., [73–75]). Furthermore, advances in sensor technology have resulted in wider availability of instruments (Table S2), enabling research groups to include these measurements in the suite of field parameters collected during field campaigns. However, limitations in cost, computing capability, and technical expertise still exist, leading to gaps in the availability of radiometric datasets (see Section 3.4.2).

In addition, ensuring that radiometric field measurements are obtained under optimal conditions continues to be challenging in coastal and inland waters. Considerations include spatiotemporal variability in illumination conditions, optical variability of the water bodies of interest, and atmospheric conditions. As such, radiometric validation data should capture: (1) different solar zenith and azimuthal angles encountered at different times of the year and/or latitudes; (2) optical variability representative of the diversity in environmental conditions within or across waterbodies; and (3) observations across different atmospheric states and aerosol influences, under varying degrees of adjacency effects. The sampling approach thus depends on the application, waterbodies of interest, and specifications of the satellite sensor(s) to be used.

Data quality can be ensured by following existing standard operating procedures, such as the ones included in the IOCCG Protocol Series [46]. Nevertheless, we should highlight that these protocols were developed for the open ocean, and hence, they pose some limitations for inland and coastal waters, as described in Section 3.2. In addition to following existing standard operating procedures, planning sampling times to be near-coincident with the satellite overpass is encouraged. Recent studies focusing on inland or coastal waters have used matchup windows of 3 h before or after the satellite overpass [55,76–79], while others selected a larger window for inland waters, often 24 h before or after the satellite overpass [80,81]. To select a reasonable temporal window, it is critical to consider the spatiotemporal dynamics in the validation location for a given system and season (see Section 3.2.1).

Furthermore, factors affecting uncertainty in radiometric field measurements should be recorded and considered. Naturally occurring changes in water constituents and illumination conditions can be accounted for in the in situ data by replicating measurements over a short period of time (in the scale of minutes, e.g., [82,83]). Systematic errors (biases) may be introduced by the sensors themselves and can be reduced by ensuring proper sensor set-up, and routine calibration and cleaning of the fore optics [45]. Basic statistics about the data, including mean, standard deviation, percentiles, and number of samples, should be reported [84]. In addition, the following ancillary data should be reported: (1) changing illumination conditions reported as cloud fraction, information on viewing geometry, and, optionally, photos of the sky in the cardinal directions; (2) potential for bottom reflectance based on the first optical or Secchi depth and water depth; (3) distance from shore to assess adjacency effects [85]; see also (Section 3.2.2); (4) sea state (wave height; surface roughness) estimated in the field or through the wind speed [86]; and (5) instrument self-shading [87,88] and shading and reflectance from the deployment platform [89,90].

Practical steps include advancing the understanding of the advantages and disadvantages of different systems and sensor set-ups for inland and coastal waters, hands-on training on sensor set-up and data acquisition, and tutorials of different data processing options and uncertainty measures. One current joint effort between EUMETSAT, NASA, and ESA is developing the Community Processor for above-water in situ data processing and uncertainty budget calculation [91] that will help the community in systematically processing hyperspectral above-water radiometry with traceable and quantifiable uncertainties. Easy and practical ways to check the calibration of sensors in the field can help identify instrument issues during data collection. More information and studies of the severity of the impacts of known issues (e.g., adjacency effects, shading, and reflectance from the deployment platform) on radiometric measurements in inland and coastal water settings and associated satellite sensors (as opposed to oceanic settings and ocean color satellite sensors) can help minimize them. Moreover, characterizing the influence of and developing corrections for illumination conditions and viewing geometries, such as the sun glint correction [92,93], on the reflectance of optically complex waters can help improve satellite retrievals for satellite remote sensing.

#### 3.1.2. Water Quality Parameters

Validation data of water quality parameters should cover the typical range of environmental variability of the water bodies of interest, both in terms of optical and biogeochemical characteristics that could affect satellite-based bio-optical models. For example, Chl *a* quantification from reflectance spectra can vary according to the composition of the algal community (e.g., differences in pigment composition and pigment packaging depending on the taxonomic group) [94]. Similarly, the composition and size distribution of particles in the water can affect the scattering and absorption of light and, consequently, the estimations of turbidity and SPM from reflectance spectra. Practically, these naturally occurring variabilities can be considered in validation datasets through: (1) the collection of data from a variety of environments and waterbodies for global validations (e.g., [61,62,95]), or (2) capturing the seasonal and inter-annual variabilities within or across waterbodies for regional validations [65,96,97]. Greater environmental variability in measurements in the dataset used for calibrating and validating bio-optical models leads to more robust performance across different environmental conditions, and a better characterization of uncertainty. The interactions between individual water quality attributes should also be considered for satellite retrievals, especially for regional assessments in highly complex and dynamic waterbodies (e.g., [98,99]). This means that simultaneous measurements of several water quality attributes might be necessary for robust validation of a given bio-optical model or algorithm.

For all parameters, it is essential to report the specifics of the methodological approach (either in situ or laboratory analysis) to characterize the uncertainty associated with the measurement [100]. For in situ sensors, including the manufacturer and model is desirable, as in situ sensor measurements can vary depending on manufacturer, calibration, and upkeep schedules (e.g., [101]). Further, an established protocol should be followed when possible and reported in the metadata. With this information, measurements that are not useful for specific studies are easier to find and discard if the measurement approach affects water quality attribute intercomparisons across different datasets.

One source of uncertainty derives from environmental variability and can be estimated from consecutive field measurements or samples over a relatively short time. Although this does not give any information regarding systematic errors (biases) (e.g., errors due to sensor calibration issues in fluorometry), replicate field measurements or sample results should be quantified and reported with average values and standard deviations or percentiles. The representativeness of these uncertainty estimations for the comparison with satellite retrievals will depend mainly on the pixel size [102] and time lag with the satellite imagery acquisition [103], and they should be considered when creating validation datasets.

In addition to the general considerations for recording and reporting ancillary information, there are considerations specific to the water quality parameter being measured. For example, when using fluorometry or spectrophotometry to measure Chl *a*, a correction may need to be made for pheophytin (see EPA methods 445.0 and 446.0 for details, [104,105]). Moreover, in vivo fluorometry of Chl *a* is susceptible to two major interferences which can introduce biases (e.g., [106]): (1) non-photochemical quenching (NPQ), which is a phenomenon by which algal fluorescence is reduced following exposure to light, and (2) inadvertent excitation of CDOM. Field fluorometry is also susceptible to the interference of suspended sediments (e.g., [107]). In the case of submerged optical sensors (e.g., turbidimeters, fluorometers), the potential interference with other instruments or the deployment structure should be evaluated and avoided. When filtering is required for laboratory measurements (e.g., SPM, CDOM), particular care should be taken regarding an appropriate pore-sized filter, especially when developing bio-optical models based on IOPs, to minimize the gap between dissolved substances and particles, as colloids may have a non-negligible contribution to scattering [108]. For Secchi depth, changing light conditions and the observer’s position relative to the sun can affect the measurements.

Some practical steps are similar to the ones already described for *R*_rs_, including the development of standard operating procedures to account for uncertainty and environmental variability, more information and studies on the severity of the impacts of known issues on water quality attribute retrievals for inland and coastal waters, and hands-on training for water quality professionals and volunteers to expand data collection. Given that field fluorometry is generally a more cost-effective and convenient way to collect data for Chl *a*, accessory algal pigments, and CDOM, the development of correction guides of known interferences for different commercial fluorometers (typically used in inland and coastal waters) would help to significantly improve the reliability of data to validate these water quality attributes. Moreover, guides on how to perform robust site-specific calibrations (e.g., for conversion from Chl *a* fluorescence to concentration) would be helpful, considering laboratory analysis error as well in the overall algorithm error budget (e.g., [109,110]).

Finally, we identify further study into the effects of particle size (algal and non-algal), composition of dissolved and particulate matter, and PCC and pigments on the absorption and scattering properties of water (i.e., on the IOPs) to better understand their effects on aquatic reflectance, and consequently, on satellite retrievals of both *R*_rs_ and water quality attributes associated with optically significant constituents.

### 3.2. From Open Ocean to Inland and Coastal Water Validation Protocols

Open ocean validation protocols have provided a strong foundation for validation efforts in inland and coastal waters (e.g., suitable validation angles and cloud cover practices, guidance for satellite pixel and field measurement matchup criteria) (e.g., IOCCG Protocol Series [46]). However, not all aspects of these protocols are applicable due to unique challenges in these systems and practical limitations (e.g., research vessels, infrastructure). Some adaptations are required for inland and coastal water validations. The following sections describe aspects of open ocean validation protocols that do not apply to inland and coastal waters, their limitations, and potential solutions.

#### 3.2.1. Time Window

Open ocean validation protocols offer a wide range of time windows to achieve coincident in situ measurements. Typically, ships-of-opportunity try to sample data near satellite overpass time, but exact temporal coincidence is not always possible as there is variability between the overpass time of different sensors (e.g., Moderate Resolution Imaging Spectroradiometer Terra (MODIS-Terra) is AM, and MODIS-Aqua is PM). The selected time window can range from 0.5 h [111] to 24 h before or after satellite overpass [81]; there are even some protocols that do not have time constraints [112]. The most common time window is 3 h before or after satellite overpass for open ocean environments [55]. These time frames aim to balance the number of matchups that can be taken and the comparability between in situ and satellite data [55].

In open ocean environments, changes in biogeochemical gradients tend to happen slowly over tens of kilometers (km). Slow gradients, coupled with generally slow current speeds in the open ocean (10^−2^ m per second), suggest that reliable matchups can be achieved hours away from the time of overpass.

Inland and coastal waters are often a dynamic mosaic of optical constituents, with significant spatiotemporal variability due to river inputs, tides, and other local forcing that can result in large biogeochemical gradients over short spatiotemporal windows. River inputs cause flushing and are themselves highly variable. The flushing moves water away from the mouth of the river, leading to mismatches in validation. The variability of river flow means that there is potential for new material to arrive between sampling and the satellite overpass, such as new vegetation that is floating downriver [113]. Similarly, regions with large tidal ranges have corresponding large currents, between 2 and 5 km/hour [114]. These currents can create substantial mismatches between observed remote sensing and in situ constituents over short time windows. Spatial variability of the system means movements can have an exaggerated effect on mismatches in the system. Validation protocols should be adjusted so that the high frequency spatial fluctuation in inland and coastal waters is accounted for in the provided time window. The difficulty here is balancing the time window with what is reasonable for sampling, being also important to identify conditions when a larger time window could be applied to increase the number of matchups [115]. Previous work looking at the effect of the time window notes that, for coastal validation, a time window closer to 0.5 h before or after satellite overpass might be more appropriate [103,116]. On the other hand, Schröder et al. [115] found that an expansion of the time window of up to 5 days before or after satellite overpass could be used for Secchi depth validation in some lakes and reservoirs. Besides these general references, we identified several factors that are important to consider when selecting the time window: tidal range (coastal waters) or mean residence time (inland waters), river discharge, current speeds, spatial variability, accessibility, and spatial resolution of the satellite sensor of interest. These factors should be considered when selecting an appropriate time window (e.g., Table S4 demonstrates how a rating system could be used to determine an appropriate time window). However, further research is needed to determine what exact sampling time window is appropriate for each of these dynamic water conditions. In addition, knowledge of the ecosystem functioning may help achieve the most optimal sampling for a given aquatic system [100]. Finally, the sensitivity of the measured parameter within the dynamic system should be considered when selecting an appropriate time window.

#### 3.2.2. Averaging and Multi-Pixel Analysis

In open ocean validation protocols, it is common practice to average a box of 9 (3 × 3) or 25 (5 × 5) pixels around the target pixel. This allows for statistics, such as the coefficient of variation, on the parameter of interest (e.g., Chl *a* or *R_rs_*) to ensure that no stray light from cloud edges, sun glint, or other artifacts are affecting the target pixel [102], and to verify that the validation region is quasi-homogeneous, to take into account errors in geocoding [55]. This works well in open ocean regions for the same reason that the large time frame works well: relatively homogeneous and slow-moving waters.

The core assumption that nearby pixels are approximately the same as the central pixel can be invalid in inland and coastal waters for several reasons. For instance, a pixel could have land in its neighboring pixels, there are often sharp gradients in optical constituents from river outflows or tidally mixed areas, and the loss of spatial resolution in inland and coastal waters makes it more difficult to validate water regions that are of human interest. As a general rule, if 3 × 3 or 5 × 5 statistics are calculated, it is crucial to ensure the regions are nearly homogeneous so that the comparison is between similar pixels [117]. Replicates of in situ measurements and samples are another approach to increase statistical confidence, as detailed in Section 3.1.2, to account for and report uncertainties. In addition, some recent studies have started to provide some guidance regarding the effects of spatial aggregation in the validation of remote sensing data products in lakes and reservoirs [115], and the spatial scales that are appropriate to adequately characterize the variability of *R_rs_* [118] or HABs [119] in certain water bodies. These types of studies are lacking across different waterbodies and for diverse water quality parameters of interest and are gaps in knowledge that can better inform remote sensing validation efforts.

#### 3.2.3. Atmospheric Correction

Open ocean validation protocols typically assume that aerosols fall on a continuous gradient and that the ocean is optically “black” at near-infrared (NIR) bands (e.g., 750 nm or 865 nm). The use of the 700 nm black assumption simplifies estimating the aerosol optical thickness by subtracting the NIR band from the rest of the spectrum to remove the atmosphere [120]. This correction method with low spatial resolution is effective for open ocean validation because the atmosphere is typically homogeneous at that scale in open ocean environments.

In inland and coastal waters, the oceanic atmospheric correction methods often perform poorly because NIR reflectance is not zero, and there are point sources that alter the atmospheric makeup. The NIR in inland and coastal waters is affected by suspended mineral particles and other optical constituents; thus, there is a non-zero NIR signal from coastal waters [121]. Atmospheric correction methods that utilize shortwave infrared (SWIR) bands can be more robust in optically complex waters [122–124], offering an improvement for inland and coastal waters; however, not all imaging sensors carry SWIR channels. For sensors that only have the NIR bands for atmospheric correction, one can use the NIR reflectance models for estimation of the NIR reflectance contributions over coastal and inland waters [125–128].

Applying a correction assuming uniform atmospheric conditions across an image or scene in open ocean environments is often useful. However, this might not work in inland and coastal waters close to human settlements, which act as point sources for changes in atmospheric makeup and optical thickness [129]. Methods that subset the scene to account for variability in atmospheric corrections might be more appropriate for these types of waters (e.g., [78,130]).

#### 3.2.4. Light Assumptions

In the open ocean, validation protocols deal with clouds and glint by masking. Many methods extend the mask by one or two pixels to remove pixels contaminated by clouds or a nearby bright body such as high glint, or sea ice [131,132]. There are other methods that make corrections for the bright body or clouds such that you can go from unaffected to affected and remove a gradient of light. This works well with the assumption that the underlying pixels are approximately the same.

Remote sensing in inland and coastal environments is frequently impacted by adjacency effects, optically shallow waters (i.e., where light reflected from the bottom contribute to *R_rs_*), and mixed/contaminated pixels, as detailed in [85]. They suggest collecting in situ data at least 5 nautical miles (~9 km) from the shore to avoid adjacency effects for ocean color validation; however, this is often neither possible nor practical for inland and coastal waters [133]. Moreover, modeling efforts show that significant adjacency effects can be seen up to 36 km offshore. These adjacency effects are up to 30% of the water signal within a few kilometers of the shore for land that is white sand and snow [85]. While these pixels can be masked, this approach often removes regions of interest. In particular, expanded masking to remove adjacency effects by two pixels prevents validation in many inland and coastal water bodies.

There is no single answer for addressing these issues, as they are highly dependent on the spatial resolution of the remote sensor and on the extent of the water body of interest. For example, sensors with spatial resolution in the order of 1 km (e.g., MODIS-Aqua, Visible Infrared Imaging Radiometer Suite (VIIRS)) are inappropriate for observing tidal channels, as a result, the information lost when masking will be less impactful. On the other hand, when using data from sensors with spatial resolutions on the order of 10–30 m (e.g., Landsat 8/9—Operational Land Imager (OLI) and OLI-2, Sentinel 2—Multispectral Imager (MSI)), masking would prevent the observation of tidal channels, which would otherwise be visible. Most contemporary atmospheric correction models have been developed using radiative transfer models that make no assumptions about adjacency effects. However, this does not mean that alternative radiative transfer modeling tools could not be employed to develop atmospheric corrections that implicitly account for straylight from land adjacent pixels. Recent efforts have been made to include physics-based corrections for adjacency effects [134]. Thus, the development of validation data sets impacted by adjacency would be beneficial so that these types of tools can be further improved to address the issue.

#### 3.2.5. Case 1 vs. Case 2 Assumptions

In open ocean environments, variability in the *R*_rs_ signal is assumed to be driven mainly by phytoplankton biomass and covarying CDOM and NAP components. This was first described by Morel and Prieur [135] as “Case 1” waters, where the optical properties of the water are attributed to phytoplankton, associated products, and the water itself. Open ocean systems are typically less optically complex because they are largely controlled by one factor. For this reason, open ocean validation protocols often only ask for pigment analysis, often high-performance liquid chromatography (HPLC), alongside IOPs and AOPs [103].

The open ocean assumption that the *R*_rs_ signal is driven by phytoplankton biomass is often invalid for inland and coastal waters (i.e., “Case 2” waters). Suspended sediments, CDOM, and pollutants, such as oil [136], agricultural runoff, and optically shallow conditions add considerable complexity to developing validation protocols for inland and coastal waters. The presence of any of these can act as a mask for the others, as at high concentrations their signals will dominate *R*_rs_ [137], making it difficult to distinguish other biogeochemical variables of interest in *R*_rs_ spectra [138]. Therefore, when sampling inland and coastal systems it is important to measure at least the three major optically active constituents: Chl *a*, SPM, and CDOM [139]. Measuring IOPs is also desirable.

### 3.3. In Situ Sensor Technologies for Measurement of IOPs

Validation of water quality parameters for inland and coastal waters represents a substantial technical challenge as existing measurement platforms, sensor technologies, and protocols have typically been developed for clearer oceanic waters. Compared to the marine environment, inland and coastal waters are often turbid, with high concentrations of scattering particles and strongly absorbing materials. In addition, concentrations of optically active constituents are often several orders of magnitude higher than in oceanic waters. Contemporary sensor technologies and deployment platforms are not always well-suited for inland and coastal waters, challenging the measurements of various parameters of interest, IOPs being among them.

#### 3.3.1. Absorption

Traditional (laboratory) methods for measuring the spectral absorption coefficients from water samples can be labor-intensive, require specialized laboratory equipment, and provide only a snapshot in time of what, for inland/coastal waters, can be highly dynamic systems. An alternative to discrete sampling is using in situ sensor technology that logs measurements continuously, such as ac-meters (Table S1). This can help characterize diurnal variability and capture rare ephemeral events.

Although ac-meters have been used by the oceanographic community for over 25 years, using ac-meters in optically complex waters has two challenges: (1) the pathlength of the flow tubes (nominally 25 cm) can be too long for highly attenuating mediums resulting in low signal-to-noise and (2) the scattering error correction [140]. Data must also be corrected for variations in pure water absorption due to temperature and salinity effects [141]. For challenge (1): while ac-meters can be manufactured with shorter flow tubes (e.g., 10 cm) to improve the signal, this still may not be short enough for highly turbid waters [142]. For challenge (2): the scattering error correction is less straightforward. The quartz sleeve of the ac-meter’s absorption tube is surrounded by an air gap, which aims to achieve total internal reflection and redirect photons scattered out of the beam path towards the detector [143]. However, a portion of photons scattered at larger angles (41–180°) are not redirected towards the detector and can lead to an overestimation of the absorption coefficient [140]. To address this, various scattering corrections have been proposed [140,144,145]. An intercomparison study by Stockley et al. [146] found that scattering corrections that use knowledge of the particulate backscattering coefficient (*b*_bp_) (e.g., [147]) or the VSF (e.g., [146]) are effective in optically complex waters; however, these approaches are contingent upon coincident measurements of *b*_bp_ or the VSF at relevant wavelengths. A more recent intercomparison study by Kostakis et al. [142] has shown that significant residual errors can remain regardless of the correction method used.

Another approach to improve in situ absorption measurements is to utilize a flow-through point source ICAM, which by design do not have the same scattering error as ac-meters [148,149]. However, sensors of this nature are, not as popular; their calibration using a liquid dye is cumbersome and their performance is not always comparable to benchtop ICAM sensors [142].

#### 3.3.2. Backscattering

The spectral particulate backscattering coefficient, *b*_bp_(*λ*), expressed in units of m^−1^, is derived from measurements of the volume scattering function (VSF, or *β*(*θ, λ*), where *θ* is the scattering angle ranging from 0–180°). Values of *b*_bp_(*λ*) can be derived from instruments that characterize *β*(*θ, λ*) in units of m^−1^ sr^−1^ [150,151] by first calculating the angular particulate volume scattering function, *β*_p_(*θ, λ*), and integrating this over the backwards direction (90–180°). Examples of commercially available sensors capable of making this measurement are listed in Table S1. These sensors may require modeling to estimate *b*_bp_(*λ*) [152,153].

Multispectral *b*_bp_(*λ*) measurements are more routinely recorded in situ using fixed angle VSF-meters [40]. Fixed angle VSF-meters measure *β*(*θ*) at a single scattering angle; then use assumptions about the shape of the particulate VSF and seawater VSF to derive *b*_bp_(*λ*) [154–156]. The relationship to convert *β*(*θ*) to *b*_bp_ relies on a non-dimensional conversion factor (*χ*(*θ*)), which is treated as independent of wavelength but dependent on scattering angle, with reported values of 1.1 and 1.08 for scattering angles of 117° and 140°, respectively [40,154]. While much research has focused on marine particle assemblages, the work by Chami et al. [157] suggested that *χ*(*θ*) not only exhibits spectral dependence but may deviate from typical values under monospecific bloom conditions, which are known to be expected in freshwater systems [158,159]. Thus, while under non-bloom conditions existing values of *χ*(*θ*) are likely acceptable, *χ*(*θ*) values for bloom events require further research and more routine measurements of *β*_p_(*θ, λ*).

It is known that commonly used backscattering sensors that have fixed gains tuned for oceanic waters (Table S1) are prone to signal saturation in highly scattering mediums [160,161]. To address this concern, novel sampling strategies have been proposed that utilize serial dilutions of in situ samples or rely on a benchtop nephelometer [161,162]. While these approaches show promise, field water samples (several liters for a dilution method) must be collected and transported to the laboratory for analysis. This approach may be suitable in some instances but may pose logistical challenges and impact temporal frequency of data. Sensors that can adjust their gain (i.e., their sensitivity or amplification level) are more likely to not saturate. Thus, thought must be given to the dynamic range requirements and the sensor capabilities, which should be carefully considered and discussed with the manufacturer.

An additional caveat of fixed-angle VSF-meters is applying a suitable correction to account for photons that are lost due to absorption and scattering along the pathlength from the source to the detection volume, and then back to the detector. Without this correction, measurements of β(θ,λ), and hence $b_{bp}(\lambda )$, will likely be underestimated. For sensors with short pathlengths, the recommended pathlength attenuation correction factor is a function of the total non-water absorption coefficient, $a_{nw}(\lambda )$ [163]. Whereas for sensors with larger dimensions and longer path lengths, the correction applied is a function of $a_{nw}(\lambda )$ and the non-water scattering coefficient, $b_{nw}(\lambda )$ [164].

A modeling study by Doxaran et al. [160] indicated that relatively short path lengths are more suitable for turbid waters, notwithstanding sensor saturation concerns, and suggested a revised version of the manufacturer’s pathlength correction. Doxaran et al. [160] also proposed an improved correction for HOBI HydroScat instruments based on $a_{nw}(\lambda )$ and $b_{nw}(\lambda )$ itself. This iterative correction was shown to be effective using IOPs and AOPs collected in optically complex waters of the Río de la Plata (South America) and Bay of Bourgneuf (France). A pathlength correction for more recent sensors in highly turbid waters is not available at the moment; however, a suitable approach would likely follow those of Doxaran et al. [160].

Existing in situ $b_{nw}(\lambda )$ measurements require coincident measurements of $a_{nw}(\lambda )$ from an ac-meter, which, in turn, requires coincident temperature and salinity data. Thus, deploying three instruments as a collective package (fixed-angle VSF, ac-meter, and temperature–salinity sensor) would be ideal to ensure all necessary data corrections that can be applied during data processing. The situation may become somewhat more complex if one opts to perform ac-meter scattering corrections that require coincident $b_{nw}(\lambda )$ and/or β(θ,λ) data, which would require an iterative approach to processing data.

Given the challenges described above to make in situ measurements of IOPs, a closure experiment can be performed by adding simultaneous in situ measurements of *R*_rs_ and relying on radiative transfer modeling. Using radiative transfer code (e.g., Numerical Optics HydroLight), it is possible to simulate *R*_rs_ using the measured IOPs and illumination conditions as inputs, and then compare it with the in situ measured *R*_rs_ spectra.

### 3.4. Current Validation Gaps

#### 3.4.1. General Trends

**Figure 2.**
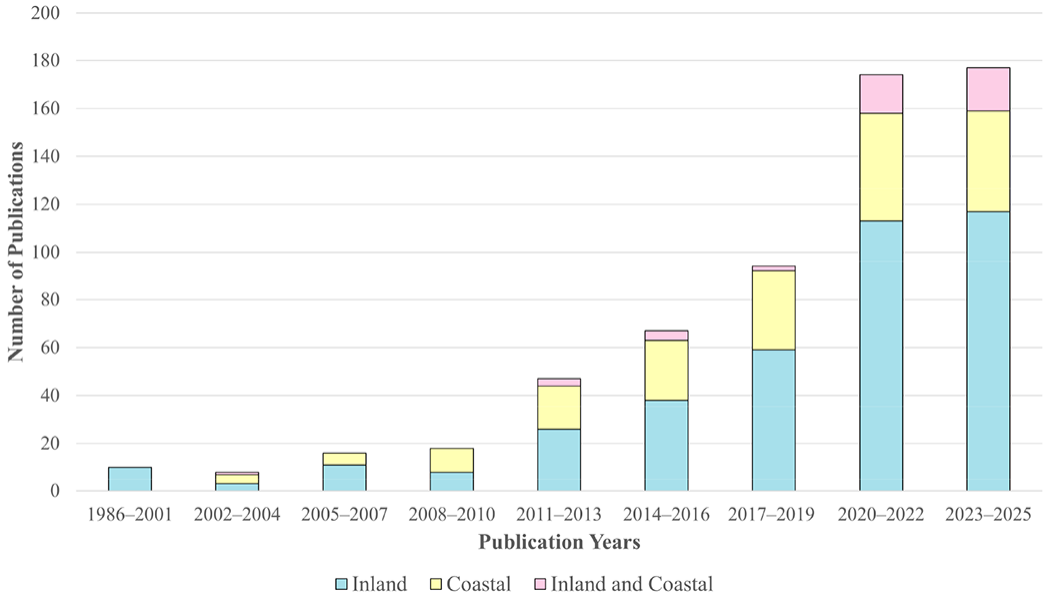
Number of published journal papers (1986–2025) on remote sensing validation studies over inland, coastal and both types of study areas.

A bibliometric analysis was performed using the Elsevier Scopus database of all publications (until 21 May 2025) to observe the general trends in validation studies over inland and coastal waters. The search was conducted based on the title, abstract, and keywords for the terms ‘remote sensing’, ‘water quality’ (including water quality parameters such as ‘Secchi Disk Depth’, ‘water clarity’, ‘chlorophyll-a’ and such), and ‘lake’ or ‘reservoir’ or ‘river’ or ‘estuary’ or ‘delta’ or ‘coastal’ or ‘inland’, and ‘validation’ or ‘evaluation.’ The results were filtered for journal papers published in English only, and only the studies validating satellite products over inland and coastal waters were selected. This resulted in 611 articles with the first one published in 1986. The number of publications for all the years is illustrated in Figure 2, which demonstrates the rapid increase in studies over the past few years.

**Figure 3.**
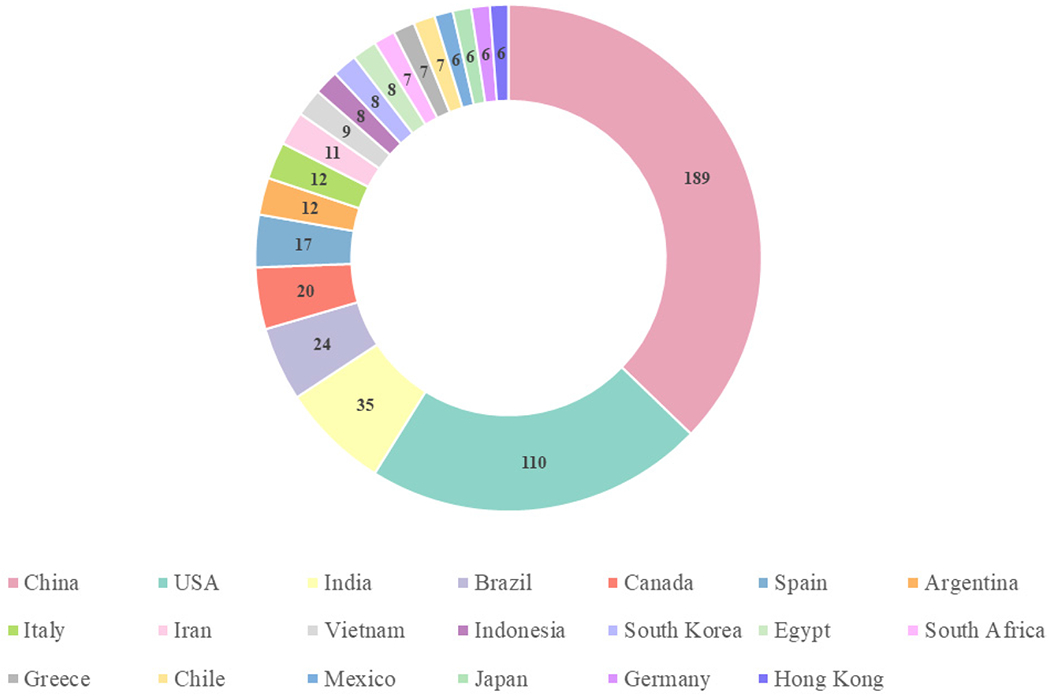
Number of inland and coastal waters remote sensing validation studies for selected countries with greater than or equal to 6 publications.

The studies were conducted over 76 countries; however, a clear geographical discrepancy between them was observed. There are 55 countries with less than 6 studies, while the remaining 20 countries account for 82% of the total studies (Figure 3). Among the top 20 countries, most studies are based in the United States and China (48.9%), which may indicate a lack of utilization of remote sensing data outside of those two countries.

#### 3.4.2. Gaps in Parameters

Historically, the widely used SeaBASS database has been primarily used to store oceanic and coastal data, including data as far back as 1930. The most common parameters found in SeaBASS are total absorption coefficient, *R*_rs_, as well as conductivity, temperature, and depth (CTD) measurements. The least frequent parameters in SeaBASS are SPM, primary productivity, phycocyanin, and total scattering coefficient [165].

The main available database for inland waters is LIMNADES, which has approximately 39,794 measurements taken from 3547 stations that are available either through request or download as of this writing. Measurements stretch back nearly 30 years, with most being recorded between 2000 and 2023. The most common parameter within LIMNADES is Chl *a* concentration (44.9%), followed by TSS (32.4%, same measurement previously referred to as SPM in the SeaBASS dataset), and *R*_rs_ (8.2%). Phycocyanin is the least common parameter (2.4%), followed by absorption coefficients for CDOM (*a*_CDOM_), phytoplankton (*a*_phy_), and non-algal particles (*a*_NAP_), with 4.5%, 4.2% and 3.3%, respectively.

Considering that for satellite validation purposes, accurate measurements of *R*_rs_ are essential (Section 3.1), it was observed that there is a lack of this type of data, especially for inland waters (only 8.2% of the data within LIMNADES). Because of the scarcity of *R*_rs_ data in inland and coastal waters, a recent effort on collecting data from both SeaBASS and individual data contributors led to the GLObal Reflectance community dataset for Imaging and optical sensing of Aquatic environments (GLORIA), which includes over 7000 hyperspectral remote sensing reflectance measurements from both inland and coastal waters [84]. In contrast, for oceanic waters, *R*_rs_ is the most abundant validation data found on combined datasets from SeaBASS, AERONET-OC, AERONET-MAN, and MOBY, whereas IOPs (*b*_bp_, *a*_CDOM_, *a*_NAP_, and *a*_phy_), *K*_d_ (490), and Chl *a* are amongst the less available validation data.

#### 3.4.3. Geographic Gaps

**Figure 4.**
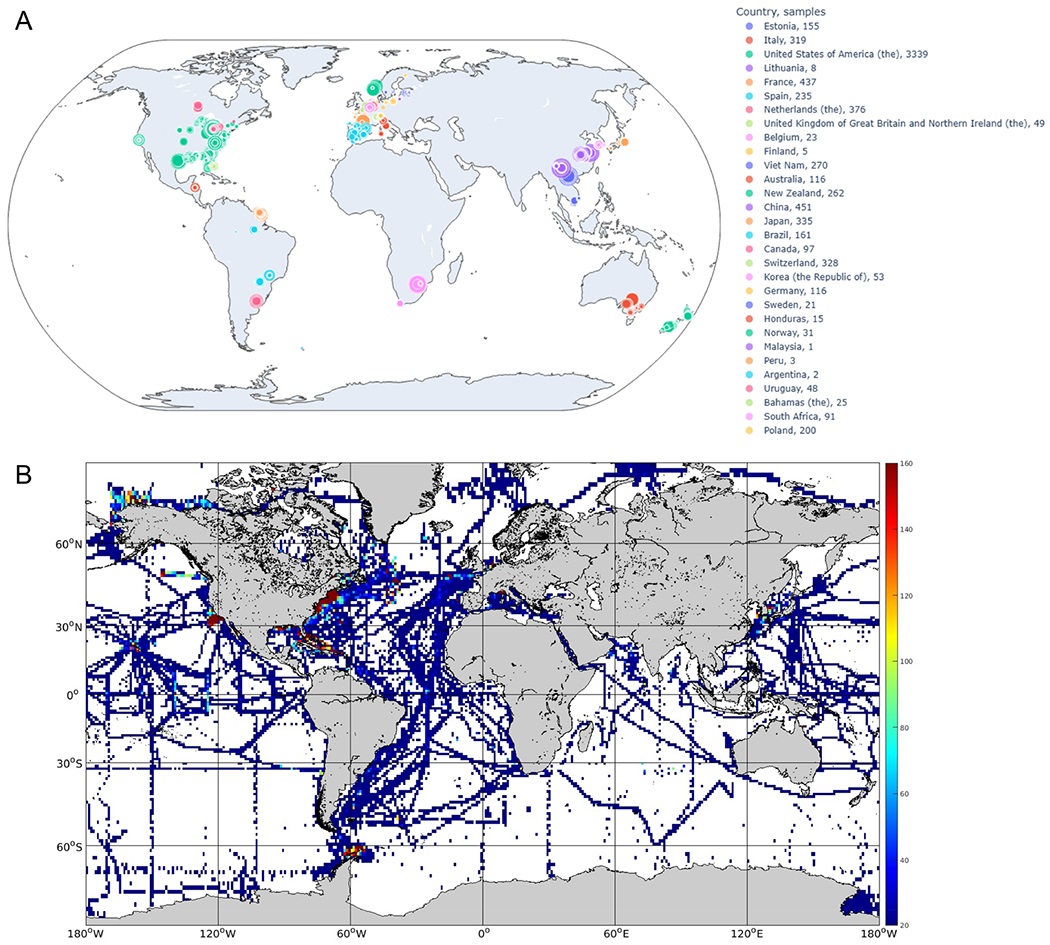
(**A**) Distribution of the data within the GLObal Reflectance community dataset for Imaging and optical sensing of Aquatic environments (GLORIA) dataset; (**B**) Relative density of SeaWiFS Bio-optical Archive and Storage System (SeaBASS) archive measurements from 1930 to 2023 based on location (shown on a 1-degree grid, provided by National Aeronautics and Space Administration SeaBASS team). Color bar indicates the number of archived projects.

While the data gaps from oceanic, coastal, and inland waters are different, there are also gaps in geographic range of data. Figure 4 shows the distribution of the data within the GLORIA dataset (Figure 4A) and the parameters of calibration and validation data in the SeaBASS database (Figure 4B). From these maps, we observe that the eastern coast of the United States is extremely well studied, as well as most of the western coast of the United States and the coast of Europe. There is a lack of data in the Indian Ocean, the Greenland Sea/Arctic Ocean, as well as the Western Pacific, Antarctic Ocean and Southeastern Atlantic (Figure 4B). For inland and coastal waters, most of the data comes from the eastern United States, western Europe, and a few areas in China, Japan, Australia, and New Zealand (Figure 4A). We observe a clear lack of data in the Global South, especially in Latin America, the Caribbean, and Africa, and in boreal high northern latitudes. These maps highlight the opportunities for future studies to support in conducting studies in these regions.

#### 3.4.4. User Engagement Gaps

A recent study about user engagement on the use of satellite data for water quality management showed that most of the participants (40.5%) self-identified as beginners with little understanding of satellite Earth observation, and 82.5% of the participants indicated that training on the use of satellite-derived data is lacking [26]. These percentages reflect the exponential growth (especially for inland waters) of the number of publications and citations in the field [166] (Figure 2), as well as the lack of training materials to support capacity building outside of the remote sensing community. There are existing training courses, such as the IOCCG Summer Lecture Series and the University of Maine “Calibration & Validation for Ocean Color Remote Sensing” (also known as the Ocean Optics Class) [167]. However, these two training opportunities are focused on the academic public, are only available in English, only offered every other year, and have limited capacity. The NASA Applied Remote Sensing Training [168] provides courses in both English and Spanish, targeting a diverse audience. It is important to provide training in different languages to avoid creating barriers for non-native English speakers. For example, Ocean Optics lectures and educational resources (e.g., [169–171]) could be translated into various languages for maximum reach and efficacy. While it may seem negligible, this advantage could disproportionately affect researchers from lower socioeconomic backgrounds, which is often associated with lower English proficiency [172]. In addition, protocols and standard operating procedures should also be translated to other languages to promote the dissemination of the content to more non-native English speaker users and ensure that everyone can collect the highest-quality data.

Besides training, including specifications regarding product development and targeted applications can help users better understand the variety of satellite-derived products and increase trust. An example is the concept of Product Family Specifications (PFS) developed by the Committee on Earth Observation Satellites (CEOS), which describes the steps taken for a product to be ready for immediate analysis, minimizes additional user effort, and are specific to the requirements of the data provider and user communities (e.g., PFS for aquatic reflectance were developed in 2022 [173]). Accompanying user success stories can also improve end-user trust by providing examples of applications of remote sensing products in a real-life context [27,174]. Furthermore, products co-developed and co-produced between scientists and end-users have been shown to provide more usable and trusted outputs [175–177].

Finally, there is a demand for the inclusion of alternative low-cost sensors to bridge the technology research gap found in some countries, especially in the Global South [178]. There is great potential with low-cost sensors to expand global validation datasets; however, adequate testing is necessary to confirm accuracy before these sensors are used for validation.

## 4. Research Opportunities

Throughout this paper we have described the current state of the science on the validation of satellite-derived data for water quality studies and monitoring in inland and coastal waters, focusing on the advances since the work of Mouw et al. [25] was published. Furthermore, we described in situ sensor technologies and databases that are available for validation of satellite products in these complex waters. We identified outstanding validation data that are still lacking to further exploit the potential of satellite sensors to better understand and manage our global water resources. Several research opportunities emerge from these validation gaps, which are described below. Additional specific considerations regarding validation of *R_rs_* , water quality attributes, and less targeted products are provided in the Supplementary Materials. Furthermore, while the focus of this paper is to highlight key in situ measurements and validation needs, the rapidly growing field of machine learning and data-driven modeling expands the areas where remote sensing models are valuable to inland and coastal water quality monitoring. These opportunities for future research include both optical and non-optical parameters (e.g., contaminants and nutrients). Additional specific research opportunities are provided in Text S1.

### 4.1. Key Research Opportunities and Considerations

1. Increased validation studies in understudied regions.
2. Development of validation educational resources that are both multilingual and written for remote sensing experts and non-experts.
3. Continued development of protocols for validation in inland and coastal waters.
4. Communication of uncertainties and expectations related to field measurements and satellite data products.
5. Further research on specific validation issues for inland and coastal waters.

Additional information on these key research opportunities is provided below:

#### 4.1.1. Validation in Understudied Regions

- Literature supports that 48.9% of validation studies are performed in the United States and China. As such, there is limited global representation of validation studies, which is probably linked to the lack of adequate training, lack of equipment, or different scientific priorities. Some regions are particularly underrepresented, including Latin America, the Caribbean, and Africa.

#### 4.1.2. Educational Resources

- Increase multilingual education of end-users so they can learn how satellite-derived products were created and their potential applications and limitations. Educational materials could include a variety of formats to account for differences in end-user capabilities (remote sensing experts and non-experts).
- Improve multilingual hands-on training for water quality professionals and volunteers to expand data collection and support training on the use of satellite-derived data for water quality management.

#### 4.1.3. Protocol Development

- Translate protocols and standard operating procedures to languages other than English to promote the dissemination of the content.
- Consider specific characteristics (e.g., optically shallow waters) when developing and/or adapting available (open ocean) protocols for measurements in inland and coastal waters.
- Collect matched data pairs of in situ reflectance and water quality attributes to help improve algorithm and model development, including the revision of implicit assumptions of open ocean models that are often adapted to inland or coastal waters.
- Record and report validation measurement metadata in a standardized format, including at least the following information: latitude, longitude, date/time, Secchi depth, water depth, elevation, wind conditions, cloud cover, and water temperature. It is also desirable to record the methods used for data collection and processing, including sensor manufacturer and model when applicable.
- Consider the appropriate sampling time-windows before and after satellite data acquisition for inland and coastal waters. The development of a time-window guide considering different characteristics of the water bodies would be very useful when designing validation sampling plans. Parameters that may be important to consider when developing a time-window guide include: tidal range (coastal waters) or mean residence time (inland waters); diurnal variability; spatial variability (homogeneous or heterogeneous); spatial resolution of the satellite sensor; and sampling accessibility (e.g., Tables S4 and S5 of the Supplementary Materials).
- Develop standard operating procedures to account for uncertainty and environmental variability of measurements. This could include but is not limited to: replication of measurements or samples over a short period of time (in the scale of minutes) to reduce and account for random errors; and report, at least, simple uncertainty quantifications, such as standard deviation, percentiles, and number of samples.

#### 4.1.4. Data Uncertainty and Expectations

- Clearly communicate uncertainties associated with both field measurements and satellite data products.
- Identify the conditions or regions for which a satellite data product is expected to perform well or poorly.
- Focus on understanding and defining which uncertainties are “acceptable” across dynamic systems through improving the understanding of end-user needs.

#### 4.1.5. Knowledge Gaps

- Severity of the impacts of known issues (e.g., adjacency effects, shading and reflectance from the deployment platform) on water quality attribute retrievals and radiometric measurements for inland and coastal waters.
- Atmospheric correction for coastal and inland waters, including: the validation of available atmospheric correction procedures across varying atmospheric and water column states to ensure robustness, the development of atmospheric corrections for inland and coastal waters that implicitly account for straylight from land adjacent pixels, and to this end, generating validation data sets impacted by adjacency effects so that tools can be generated to further address the issue.
- Effects of particle size (algal and non-algal), composition of dissolved and particulate matter, and algal community composition and pigments on the absorption and scattering properties of inland and coastal water bodies (i.e., on the IOPs) to better understand their effects on aquatic reflectance.
- Improving in situ absorption and scattering sensor designs or developing corrections for existing sensors that work well in highly attenuating/scattering mediums, which are often common in inland/coastal waters and incorporate these measurements in field campaigns.
- Characterization of known interferences and issues (e.g., NPQ, temperature quenching, etc.) with in situ fluorometry-based sensors (e.g., Chl *a*, CDOM, accessory algal pigments) to expand their use as satellite validation data.

## Supplementary Material

Supplement1

The following supporting information can be downloaded at: https://www.mdpi.com/article/10.3390/rs17244008/s1, Table S1: Examples of currently available sensors to measure IOPs, including pros and cons for each type of sensor; Table S2: Examples of sensors used to measure AOPs, including pros and cons for each sensor; Table S3: Examples of sensors used to measure various water quality parameters, including pros and cons for each sensor type; Table S4: Example table to evaluate the appropriate time frame for validation of satellite retrievals in coastal waters (please note that values presented here are shown as examples and not supported by peer-reviewed research); Table S5: Example table to select an appropriate time frame for validation of satellite retrievals in coastal waters (please note that values presented here are shown as examples and not supported by peer-reviewed research); Text S1: Additional Specific Research Opportunities.

## Figures and Tables

**Table 1.** Non-exhaustive list of current space agency-led satellite-based remote sensing missions with publicly available data. All missions are operational as of 21 May 2025. Spatial, spectral, and temporal resolution specifications were compiled from space agency websites. Sensor name, satellite name, space agency and country are provided, including websites where more information on the mission can be obtained, as well as links to data access.

Sensor	Satellite Agency Region	Orbit	Spatial Resolution (m)	Spectral Resolution (nm)	Temporal Resolution	Website
AHI	HIMAWARI-8 JMA Japan	Geostationary	1000–2000	470–1331 (16 bands)	10 min	Meteorological Satellite Center (MSC)|HOME (https://www.data.jma.go.jp/mscweb/en/index.html)
AHI	HIMAWARI-9 JAXA Japan	Geostationary	1000–2000	470–1331 (16 bands)	10 min	Meteorological Satellite Center (MSC)|HOME (https://www.data.jma.go.jp/mscweb/en/index.html)
GOCI-II	GeoKompsat-2B KARI/KIOST South Korea	Geostationary	250	380–900	10 times per day	Korea Ocean Satellite Center (https://kosc.kiost.ac.kr/index.nm?menuCd=44&lang=en)
HYC	PRISMA ASI Italy	Polar	30	400–1010 (hyperspectral, 66 bands), and 920–2505 (hyperspectral, 173 bands)	User-defined targets	ASI|Agenzia Spaziale Italiana (https://www.asi.it/en/earth-science/prisma/)
MSI	Sentinel-2A/B/C ESA EU	Polar	10–20–60	442–2202 (13 bands)	10 days	Sentinel-2|Copernicus Data Space Ecosystem (https://dataspace.copernicus.eu/data-collections/copernicus-sentinel-data/sentinel-2)
MODIS	Aqua (EOS-PM1) NASA USA	Polar	250–1000	405–2130 (13 bands)	1 day	MODIS Web (https://modis.gsfc.nasa.gov/)
MODIS	Terra (EOS-PM2) NASA USA	Polar	250–1000	405–2130 (13 bands)	1 day	MODIS Web (https://modis.gsfc.nasa.gov/)
OCI	PACE NASA USA	Polar	1000	317–885, 1240–2250 nm (hyperspectral, 5 nm spacing)	1 day	NASA PACE - Home (https://pace.gsfc.nasa.gov/)
OCM	Oceansat-2 ISRO India	Polar	360 × 236	404–885 (8 bands)	2 days	Oceansat-2 (https://www.isro.gov.in/Oceansat_2.html)
OCM	EOS-6-Oceansat-3 ISRO India	Polar	360/1080	412–1010 (13 bands)	2 days	EOS-06 (https://www.isro.gov.in/EOS_06.html)
EnMAP	Environmental Mapping and Analysis Program DLR-EOC Germany	Polar	30	420–1000 (hyperspectral 6.5 nm spacing) 900–2450 (hyperspectral 10 nm spacing)	27 days	EnMAP (https://www.enmap.org/)
PMC-2	Gaofen-2 CRESDA China	Polar	Sub-meter	Multispectral (4 bands VIS-NIR)	5 days	Earth Observation Satellites from CRESDA (https://database.eohandbook.com/database/agencysummary.aspx?agencyID=130)
OLCI	Sentinel-3A/B ESA/EUMETSATEU	Polar	300	400–1020 (16 bands)	2 days	Sentinel-3|EUMETSAT (https://www.eumetsat.int/sentinel-3)
OLI	LandSat-8 NASA/USGS USA	Polar	30	442–2200 (9 bands)	16 days	Landsat 8|U.S. Geological Survey (https://www.usgs.gov/landsat-missions/landsat-8)
OLI-2	LandSat-9 NASA/USGS USA	Polar	30	442–2200 (9 bands)	16 days	Landsat 9|U.S. Geological Survey (https://www.usgs.gov/landsat-missions/landsat-9)
SGLI	GCOM-C JAXA Japan	Polar	250–1000	375–12,500 (19 bands)	2–3 days	JAXA|Global Change Observation Mission - Climate “SHIKISAI” (GCOM-C) (https://global.jaxa.jp/projects/sat/gcom_c/)
VIIRS	Suomi NPP NOAA USA	Polar	375/750	412–11,800 (22 bands)	1 day	Joint Polar Satellite System|NESDIS|National Environmental Satellite, Data, and Information Service (https://www.nesdis.noaa.gov/our-satellites/currently-flying/joint-polar-satellite-system)
VIIRS	JPSS-1/NOAA-20 NOAA/NASA USA	Polar	375/750	412–11,800 (22 bands)	1 day	Joint Polar Satellite System|NESDIS|National Environmental Satellite, Data, and Information Service (https://www.nesdis.noaa.gov/our-satellites/currently-flying/joint-polar-satellite-system)
VIIRS	JPSS-2/NOAA-21 NOAA/NASA USA	Polar	375/750	412–11,800 (22 bands)	1 day	Joint Polar Satellite System|NESDIS|National Environmental Satellite, Data, and Information Service (https://www.nesdis.noaa.gov/our-satellites/currently-flying/joint-polar-satellite-system)
COCTS	HY-1C/1D NSOAS/MNR China	Polar	1000	412–1200 (10 bands)	1 day	HY-1C/1D (HaiYang-1C/1D) - eoPortal (https://www.eoportal.org/satellite-missions/hy-1c-1d#eop-quick-facts-section)
CZI	HY-1C/1D NSOAS/MNR China	Polar	50	460–825 (5 bands)	1 day	HY-1C/1D (HaiYang-1C/1D) - eoPortal (https://www.eoportal.org/satellite-missions/hy-1c-1d#eop-quick-facts-section)

**Table 2.** Summary of acronyms and symbols used in this review.

Symbol/Acronym	Definition
^*a*^	Absorption coefficient
^*a*^_CDOM_	Absorption coefficient of CDOM
^*a*^_NAP_	Absorption coefficient of non-algal particles
^*a*^_nw_	Non-water absorption coefficient
^*a*^_p_	Absorption coefficient of suspended particles (phytoplankton + NAP)
^*a*^_phy_	Absorption coefficient of phytoplankton
AOP	Apparent Optical Property
^*b*^	Scattering coefficient
^*b*^_b_	Backscattering coefficient
^*b*^_bp_	Backscattering coefficient of suspended particles
^*b*^_nw_	Non-water scattering coefficient
c	Beam attenuation
CDOM	Chromophoric Dissolved Organic Matter
Chl *a*	Chlorophyll *a* concentration
DOC	Dissolved Organic Carbon
*E*_d_	Downwelling irradiance
HAB	Harmful Algal Bloom
HPLC	High-Performance Liquid Chromatography
IOP	Inherent Optical Property
*K*_d_	Diffuse attenuation coefficient of downwelling irradiance
*K*_PAR_	Diffuse attenuation coefficient of photosynthetically active radiation
*L*_w_	Upwelling (water-leaving) radiance
NAP	Non-Algal Particles
NIR	Near-Infrared
NPQ	Non-Photochemical Quenching
PC	Phycocyanin
PCC	Phytoplankton Community Composition
PE	Phycoerythrin
*R*_rs_	Remote sensing reflectance
SPM/TSS	Suspended Particulate Matter/Total Suspended Solids
SWIR	Shortwave Infrared
VIS	Visible spectrum
VSF or *β*	Volume scattering function
*β*_p_	Particulate volume scattering function

## Highlights

### What are the main findings?

- Review of the state of science of satellite-derived optical and water quality products.
- Field data measurement review for remote sensing validation in inland and coastal waters.

### What is the implication of the finding?

- Guidance for the scientific community to consider when implementing field campaigns to collect data for remote sensing validation needs.
