# Supplementary material for: Advancements in Satellite Observations of Inland and Coastal Waters: Building Towards a Global Validation Network
Source: Remote Sens (Basel). Author manuscript; Available in PMC 2026 Mar 11. (PMC12973240; doi:10.3390/rs17244008)
Supplement: Supplement1 [file NIHMS2130632-supplement-Supplement1.pdf]

Advancements in Satellite Observations of Inland and Coastal Waters: Building towards a Global Validation Network

Dulcinea M. Avouris [1,*,†], Fernanda Maciel [2,†], Samantha L. Sharp [3,4,†], Susanne E. Craig [5,6], Arnold G. Dekker [7], Courtney A. Di Vittorio [8], John R. Gardner [9], Emma Goldsmith [11,12], Juan I. Gossn [13], Steven R. Greb [14], Brice K. Grunert [15], Daniela Gurlin [16], Mahesh Jampani [17], Rabia Munsaf Khan [18], Ben Lowin [10], Lachlan McKinna [5,19], Colleen B. Mouw [20], Igor Ogashawara [21], Sara RiveroCalle [10], Wilson Salls [22], Joan-Albert Sánchez-Cabeza [23], Blake Schaeffer [24], Bridget N. Seegers [5], Jari Silander [25], Emily A. Smail [26], Menghua Wang [27] and Jeremy Werdell [5]

1 U.S. Geological Survey, California Water Science Center, Sacramento, CA 95819, USA
2 Instituto de Mecánica de los Fluidos e Ingeniería Ambiental, Facultad de Ingeniería, Universidad de la República, Montevideo 11300, Uruguay; fmaciel@fing.edu.uy
3 Tahoe Environmental Research Center, University of California, Davis, CA 95616, USA; ssharp@ucdavis.edu
4 NASA Ames Research Center, Moffett Field, CA 94035, USA
5 NASA Goddard Space Flight Center, Greenbelt, MD 20771, USA; susanne.e.craig@nasa.gov (S.E.C.); lachlan.mckinna@go2q.com.au (L.M.); bridget.n.seegers@nasa.gov (B.N.S.); jeremy.werdell@nasa.gov (J.W.)
6 Goddard Earth Sciences Technology and Research II, University of Baltimore County, Baltimore, MD 21228 Baltimore, MD 21251, USA
7 CSIRO (Commonwealth Scientific Industrial Research Organisation), Canberra, ACT 2601, Australia; arnoldgdekker@gmail.com
8 Engineering Department, Wake Forest University, Winston-Salem, NC 27101, USA; divittoc@wfu.edu
9 Department of Geology and Environmental Science, University of Pittsburgh, Pittsburgh, PA 15260, USA; gardner.john@pitt.edu
10 Skidaway Institute of Oceanography, University of Georgia, Savannah, GA 31411, USA; ben.lowin@uga.edu (B.L.); rivero@uga.edu (S.R.C.)
11 Department of Environmental Science, Creighton University, Omaha, NE 68131, USA
12 BAE Systems Inc. Space & Mission Systems, Boulder, CO 80301, USA; Emma.goldsmith@baesystems.us
13 European Agency for the Exploitation of Meteorological Satellites, 64295 Darmstadt, Germany; juancho.gossn@gmail.com
14 Cooperative Institute for Meteorological Satellite Studies, University of Wisconsin-Madison, Madison, WI 53706, USA; srgreb@wisc.edu
15 Department of Biological, Geological, and Environmental Sciences, Cleveland State University, Cleveland, OH 44115, USA; b.grunert@csuohio.edu
16 Wisconsin Department of Natural Resources, Madison, WI 53707, USA; daniela_gurlin@hotmail.com
17 International Water Management Institute (IWMI-CGIAR), Colombo 10120, Sri Lanka; m.jampani@cgiar.org
18 College of Environmental Science and Forestry, State University of New York, Syracuse, NY 13210, USA; rkhan06@syr.edu
19 GO2Q PTY LTD, North Rocks, NSW 2151, Australia
20 Graduate School of Oceanography, University of Rhode Island, Narragansett, RI 02882, USA; cmouw@uri.edu
21 Department of Plankton and Microbial Ecology, Leibniz Institute of Freshwater Ecology and Inland Fisheries, 16775 Stechlin, Germany; igor.ogashawara@igb-berlin.de
22 U.S. Geological Survey, New York Water Science Center, Troy, NY 12180, USA; wsalls@usgs.gov
23 Unidad Académica Mazatlán, Instituto de Ciencias del Mar y Limnología, Universidad Nacional Autónoma de México, Mazatlán 82040, México; jasanchez@cmarl.unam.mx
24 U.S. Environmental Protection Agency, Office of Research and Development, Durham, NC, 27709 USA; schaeffer.blake@epa.gov
25 Finnish Environment Institute, 00790 Helsinki, Finland; jari.silander@syke.fi

Academic Editor(s): Name

Received: 5 September 2025
Revised: 1 December 2025
Accepted: 3 December 2025
Published: date

Citation: Avouris, D.M.; Maciel, F.; Sharp, S.L.; Craig, S.E.; Dekker, A.G.; Di Vittorio, C.; Gardner, J.R.; Goldsmith, E.; Gossn, J.I.; Greb, S.R.; et al. Advancements in Satellite Observations of Inland and Coastal Waters: Building Towards a Global Validation Network. *Remote Sens.* **2025**, *17*, x. https://doi.org/10.3390/xxxxx

Copyright: © 2025 by the authors. Submitted for possible open access publication under the terms and conditions of the Creative Commons Attribution (CC BY) license (https://creativecommons.org/licenses/by/4.0/).

[26] NOAA/NESDIS/Office of Satellite and Product Operations, College Park, MD 20740, USA; emily.smail@noaa.gov
[27] NOAA Center for Satellite Applications and Research, College Park, MD 20740, USA; menghua.wang@noaa.gov
* Correspondence: dulceavouris@gmail.com
† These authors contributed equally to this work.

Supplementary Material

Any use of trade, firm, or product names is for descriptive purposes only and does not imply endorsement by the U.S. Government.

Table S1. Examples of currently available sensors to measure IOPs, including pros and cons for each type of sensor.

Sensor types	Example Sensor	Pros	Cons
Absorption (a)	Absorption and attenuation sensors (ac-meters) (e.g., Sea-Bird Scientific ac-s) or absorption sensors (e.g., TriOS OSCAR)	Sensors can be used for laboratory and/or in-situ absorption and/or attenuation measuring including profiling<br><br>Sensors can be calibrated for water quality attribute measuring<br><br>ac-s is a spectrophotometer providing hyperspectral absorption and attenuation whereas OSCAR is a flow-through online hyperspectral integrating cavity absorption meter providing accurate absorption	Sampling location specific calibrations can be needed for water quality attribute measuring<br><br>Different instrument designs and approaches to account for scattering can lead to different measurement accuracies<br><br>Pathlengths optimized for oceanic waters may be too long for turbid waters resulting in low signal.<br><br>Degassing OSCAR in situ can be challenging
Backscatter (Bb)	Scattering sensors<br>(e.g., Sea-Bird Scientific ECO and ECO Puck, Sequoia Scientific Hyper-bb, IMO SC6 (all used for bb measuring)), Sequoia LISST-VSF	Sensors can be used for bbp measuring representative of particle abundance<br><br>ECO Triplet sensors can be customized for bb, bb-based, and/or fluorescence-based water quality attribute measuring in up to three channels<br><br>ECO Puck sensors are specifically designed for use in AUVs, profiling floats, and Slocum gliders<br><br>Hyper-bb can be used for hyperspectral bb measuring and addresses the limited spectral coverage of existing bb sensors	Default gains of ECO sensors can lead to channel saturation in some waters<br><br>Sampling location specific range adjustments or calibrations can be needed for bb- and fluorescence-based water quality attribute measuring<br><br>Some sensor calibrations are challenging and sensors used for bb measuring are typically serviced at the manufacturers<br><br>LISST-VSF is single wavelength
Beam attenuation (c)	Absorption and attenuation sensors (e.g., Sea-Bird Scientific ac-s) or transmission and attenuation sensors (e.g., TriOS	Sensors can be used for laboratory and/or in-situ absorption and/or attenuation measuring including profiling	Different instrument designs including different fields of view (FOV) and approaches to account for scattering at angles smaller than the FOV can lead to different measurement accuracies

	VIPER, Sea-Bird Scientific C-Star, and C-Rover, Sequoia Scientific LISST-Tau)	ac-s is a spectrophotometer providing multispectral absorption and attenuation whereas VIPER is a VIS transmissometer providing hyperspectral attenuation<br><br>Other sensors include transmissometers targeting single wavelength transmission and/or attenuation	

Table S2. Examples of sensors used to measure AOPs, including pros and cons for each sensor.

Sensor types	Example Sensor	Pros	Cons
Reflectances (e.g., R_{rs})	Multispectral radiance and irradiance sensors (e.g., BSI C-OPS and XRR (for profiling), CIMEL CE318-TV12-OC and LC (for automated above-water measuring))	Sensors have consolidated technology, are generally little affected by stray light and polarization, and use reliable optical-filter technology<br><br>Sensor bands are distributed across a spectral range<br><br>C-OPS is a radiometer system providing simultaneous in-water radiance and irradiance in 19 spectral bands and can be used for shallow-water profiling<br><br>CE318-TV12-OC and LC (SeaPRISMs for OceanColor and LakeColor) are automated photometers providing sequential above-water radiance and irradiance using 12-filter optical-filter wheels and can be used for fixed station measuring	Number of spectral bands is limited<br><br>Sensor bands can need to be shifted to satellite sensor central bands in the data processing<br><br>Proprietary data acquisition and processing software<br><br>Sensors are typically serviced at the manufacturer
	Hyperspectral radiance and irradiance sensors<br>(e.g., Sea-Bird Scientific HyperOCR, TriOS RAMSES, IMO USSIMO(for all types of measuring), ASD FieldSpec 4, SVC HR and XHR, Spectral Evolution PSR+, PSR1100f and SR-3500, Ocean Insight SR4, AvaSpec CompactLine (for above-water and near-surface in-water measuring (via fiber optics)), Water Insight WISP-3 (for above-water measuring) and WISPstation (for automated above-water measuring)) and IMO DALEC and SBE HyperSAS (for above-water)	Sensors have consolidated technology, are generally little affected by stray light and polarization, and use reliable optical-filter technology<br><br>Sensor bands are distributed across a spectral range<br><br>Number of spectral bands is expansive<br><br>Sensor bands can be matched to satellite sensor central bands in the data processing<br><br>HyperOCR and RAMSES are radiometers with a fully characterized cosine response providing accurate radiance or irradiance for all types of measuring whereas most others are associated with specific types of measuring<br><br>DALEC and HyperSAS have pointing capabilities to ensure optimal geometry is maintained.	Sensors can lack a full optical characterization including straylight analysis and characterization<br><br>Proprietary data acquisition and processing software<br><br>Sensors are typically serviced at the manufacturer<br><br>Deployment strategies must avoid self-shading.<br><br>Single spectrometer measurements require a spectralon reflective plaque which must be kept clean.

Diffuse attenuation coefficients (K-functions, e.g., Kd)	Multi- or hyperspectral radiance and irradiance, or quantum sensors (e.g., BSI C-OPS, XRR, and QCP, Sea-Bird Scientific HyperOCR and ECO PAR, TriOS RAMSES, LI-COR Biosciences LI-192, YSI EXO PAR System with LI-192 sensors, IMO USSIMO, IMO MS9)	Sensors used for radiance and irradiance measuring in profiling applications can provide the corresponding diffuse attenuation coefficients from the same data<br><br>Underwater quantum sensors are specialized sensors and can provide PAR and its diffuse attenuation coefficients using silicon photodiodes and optical-filter technology to create a uniform sensitivity to light over wavelengths from 400 to 700 nm<br><br>Underwater quantum sensors are routinely used in water quality monitoring programs and can provide a simple measurement of underwater light availability	Sensors are typically serviced at the manufacturer

Table S3. Examples of sensors used to measure various water quality parameters, including pros and cons for each sensor type.

Sensor types	Example Sensor	Pros	Cons
Turbidity	NIR b_b-based turbidity sensors (e.g., Sea-Bird Scientific ECO and ECO Puck)	Sensors are commonly used for nearshore and open ocean water quality monitoring<br><br>Sensors can be used for b_b-based turbidity measuring providing an alternative to 90° IR scattering-based turbidity sensors<br><br>ECO sensors can be customized for b_b, b_b-based, and/or fluorescence-based water quality attribute measuring in up to three channels<br><br>ECO Puck sensors are specifically designed for use in AUVs, profiling floats, and Slocum gliders	Default calibrations of ECO sensors can lead to channel saturation in some waters<br><br>Sampling location specific range adjustments or calibrations can be needed for b_b- and fluorescence-based water quality attribute measuring<br><br>Comparability to turbidity measurements using 90° IR scattering-based turbidity sensors can be limited
	90° IR scattering-based turbidity sensors (e.g., TriOS TTurb, Eureka Water Probes Trimeter and Manta+, Turner Designs C3, C6P, C-FLUOR, and Cyclops-7F, YSI EXO, ProDSS, and ProSwap + probes/sensors)	Sensors are commonly used for inland water quality monitoring<br><br>Sensors adhere to national and international standards for turbidity measuring<br><br>Multiparameter probes find widespread use and can include temperature, depth, turbidity, fluorescence, and other sensors<br><br>Individual and multiparameter probes can be used with *in-situ* deployment options ranging from hand-held sampling and profiling to autonomous profiling and mooring	Comparability to turbidity measurements from NIR b_b-based turbidity sensors can be limited
Particle size distribution	Particle size analyzers (e.g., Sequoia Scientific LISST-200X and LISST-HAB)	Sensors are commonly used in ocean optics<br><br>LISST-200X can be used for particle size, concentration, beam attenuation, VSF, depth, and temperature measuring	Sensors might exceed practical needs for most satellite data validation studies in inland waters in terms of measured IOPs and water quality attributes and associated costs<br><br>VSF is measured at a single wavelength

		LISST-HAB is based on the LISST-200X and integrates Turner Designs Cyclops-7F fluorometers for additional PC, PE, and Chl*a* measuring<br><br>Sensors can be used with different *in-situ* deployment options including complementing CTDs	
Chl*a*, PC, PE, and CDOM	Absorption and attenuation sensors (e.g., Sea-Bird Scientific ac-s), absorption sensors (e.g., TriOS OSCAR), or attenuation sensors (e.g., TriOS VIPER)	Sensors can be used for laboratory and/or in-situ absorption and/or attenuation measuring including profiling<br><br>Sensors can be calibrated for water quality attribute measuring<br><br>Sensors can be used for many water quality attributes beyond b_b- and fluorescence-based sensors including for NO3-N, TOC, and DOC measuring<br><br>Some sensors are adapted to online water quality monitoring	
	Fluorescence sensors<br>(e.g. Sea-Bird Scientific ECO and ECO Puck, TriOS matrixFlu VIS and nanoFlu,<br>Eureka Water Probes Trimeter and Manta+, Turner Designs C3, C6P, C-FLUOR, and Cyclops-7F, YSI EXO + probes/sensors, bbe Moldaenke FluoroProbe and PhycoProbe)	Sensors are commonly used from nearshore and open ocean to inland water quality monitoring<br><br>Sensors can be used for Chl*a*, PC, PE, CDOM, fluorescence and<br>rhodamine (tracing applications) measuring<br><br>Sensors can be re-calibrated by users via software if sampling location specific<br>calibrations are desired for increased accuracy<br><br>Re-calibrations of most sensors are more straightforward than for absorption and attenuation sensors<br><br>Some individual and multiparameter probes can be used in wired or wireless configurations	

Table S4. Example table to evaluate the appropriate time frame for validation of satellite retrievals in coastal waters (please note that values presented here are shown as examples and not supported by peer-reviewed research).

	**Tide range (m)**	**River source**	**Current speeds (m/s)**	**Spatial variability**	**Accessibility**	**Satellite spatial resolution (km)**
**1**	<0.5	Low	<0.2	Homogenous	Difficult	>1
**2**	0.5-1.5	Medium	0.2-0.8	Mixed	Moderate	1-0.1
**3**	>1.5	High	>0.8	Heterogeneous	Easy	<0.1
**Score**						

Table S5. Example table to select an appropriate time frame for validation of satellite retrievals in coastal waters (please note that values presented here are shown as examples and not supported by peer-reviewed research).

**Total Score**	**Suggested time frame (+/- hr)**
6-9	3
10-13	2
14-18	1

Text S1: Additional Specific Research Opportunities

1. Validating R_{rs}

Based on current databases, there is a clear lack of validation data of R_{rs} for inland and coastal waters. To promote the validation of R_{rs}, we suggest:

- Researching the advantages and disadvantages of different system and sensor set-ups and associated uncertainties.
- Promoting radiometric measurements as standard environmental monitoring parameters by:
  - Development of more cost-effective sensor technologies.
  - Developing training materials (guides, videos, etc.) for data acquisition, data processing and uncertainty measures.
  - Developing easy and practical ways to check the calibration of sensors in the field
- Promoting awareness to the community of the occurrence of instrument characteristics such as non-linearity of response, straylight, angular response, among others, and the need to account for such effects in the uncertainty budget of measurements - and if possible, correct for them.

Specifically for R_{rs}, *in situ* validation data would benefit from:

- Capturing different solar zenith and azimuthal angles encountered at different times of the year and/or latitudes.
- Capturing the optical variability representative of the diversity in environmental conditions within or across waterbodies.
- Capturing different atmospheric states and aerosol influences under varying degrees of adjacency effects.
- Prioritizing data quality over quantity, minimizing the time difference between data collection and satellite overpass.
- Ensuring proper sensor set-up and calibration to reduce systematic biases, although further studies of the specific needs for inland and coastal waters are needed.
- Simultaneously recording and reporting information to assess known interfering conditions and issues:
  - For illumination conditions: cloud fraction, viewing geometry, and (optionally) photos of the sky in the cardinal directions.
  - For potential bottom reflectance: first optical depth or Secchi depth and water depth. For potential adjacency effects: distance from nearest shore.
  - For the air-water interface: wave height and surface roughness.
  - For potential instrument/sensor shading: instrument type, viewing geometry, platform specifics, and distance from the platform or other structures.

2. Validating water quality attributes

This section refers mainly to parameters that are more commonly or traditionally measured in environmental monitoring of inland and coastal waters. Hence, to expand the validation of water quality attributes, we suggest:

Specifically for water quality attributes, in situ validation data would benefit from:

- Covering the typical range of environmental variability of the water bodies of interest. Practically, this means collecting data from a variety of environments and waterbodies for global validations or capturing the seasonal and inter-annual variability within or across waterbodies for regional validations.
- Considering the interactions between different water constituents for a robust validation of bio-optical models or algorithms. Measuring the three major optically active constituents in inland and coastal waters is essential: Chl *a*, SPM, and CDOM.
- Reporting the specifics of the measurement approach to characterize the uncertainty associated with it, including the sensor manufacturer and model.
- Following an established protocol, when possible, and report it in the metadata.
- Ensuring proper sensor/instrument calibration, including site-specific calibration when needed (for example, for *in situ* fluorometry), and correction of measurements for interfering conditions (e.g., for pheophytin when using laboratory spectrophotometry or fluorometry to measure Chl *a*; NPQ, and CDOM fluorescence for *in situ* fluorometry of Chl *a*, etc.).

3. Validating less targeted products

We believe promoting the characterization of (all) optically active constituents is important to understand the dynamics of inland/coastal aquatic systems and associated water reflectance spectra. Ideally, this includes collecting data of IOPs, PCC, and identifying cyanobacteria species (responsible for freshwater HABs). However, *in situ* sensor technologies to measure IOPs in inland and coastal waters present important limitations, as described in Section 3.4. Therefore, for an appropriate validation of IOPs, we suggest:

- Improving/developing *in situ* sensor designs to measure absorption and scattering coefficients that work well in highly attenuating/scattering mediums, which are often common in inland and coastal waters. Sensor capabilities can be carefully considered and discussed with the manufacturer.
- Meanwhile, when using available ac meters (to derive absorption coefficients), ensuring appropriate length of the flow tube (although not always possible), and appropriate scattering correction, although significant residual errors can remain regardless of the correction. Likewise, when using available b_b meters (to derive backscattering coefficients), ensuring proper pathlength correction, although further analyses/research of these corrections are needed for some of the commercial sensors.
- Conducting more research and measurements of estuarine, coastal, and freshwater particle assemblages to derive backscattering coefficients from *in situ* measurements at a single scattering angle (e.g., under near-monospecific bloom conditions).
- Carefully considering the co-deployment of several instruments to ensure all necessary data corrections can be applied during data processing, as correction

procedures often need coincident data of several IOPs. In addition, comparing modeled (using measured IOPs and a radiative transfer model) with *in situ* measurements of R_{rs} is desirable as a "closure experiment", in which good agreement indicates accuracy in measured IOPs.
